# Donor Time to Death and DCD Liver Transplant Outcomes: Challenging the Dogma That Shorter Is Better

**DOI:** 10.1097/TXD.0000000000001911

**Published:** 2026-02-11

**Authors:** Dharesh Raj Amarnath, Samuel J. Tingle, Georgios Kourounis, Abdullah K. Malik, Garrett R. Roll, Chris Freise, Seiji Yamaguchi, Charles Rickert, Colin H. Wilson

**Affiliations:** 1 Newcastle University School of Medicine, Newcastle upon Tyne, United Kingdom.; 2 National Institute of Health and Care Research Blood and Transplant Research Unit, Newcastle University and Cambridge University, Newcastle upon Tyne, United Kingdom.; 3 Institute of Transplantation, Freeman Hospital, Newcastle upon Tyne, United Kingdom.; 4 Translational and Clinical Research Institute, Newcastle University, Newcastle upon Tyne, United Kingdom.; 5 Department of Surgery, Division of Transplantation, University of California San Francisco, San Francisco, CA.

## Abstract

**Background.:**

In circulatory death transplantation, time to death (TTD) following withdrawal of life-sustaining treatment is unpredictable. Concerns persist that prolonged TTD may cause ischemic injury, resulting in organ nonuse. We assessed the impact of donor TTD on liver transplant outcomes and utilization.

**Methods.:**

We used Organ Procurement and Transplantation Network data on adult donors after circulatory death transplants (2010–2025). Multivariable nonlinear (restricted cubic splines) regression models were used to analyze associations. Simulation studies estimated potential increases in liver acceptance rates.

**Results.:**

In 8489 recipients, short donor TTD was associated with inferior outcome, contrary to popular belief. Prolonged TTD did not show inferior posttransplant outcomes, irrespective of whether normothermic regional perfusion (NRP) was used. Nonlinear modeling (n = 37 447) revealed a sharp decline in utilization once TTD exceeded just 15 min. Given prolonged TTD did not impact outcomes, these declines represent a missed opportunity for organ use. Simulation studies revealed that if surgeons assess organ offers from patients with TTD of 15–30 min identically to those with TTD <10 min, there would have been a 17.1% (95% confidence interval, 15.0%-19.3%) relative increase in utilization, with potentially better outcomes than current practice. In the setting of NRP, short TTD was associated with increased organ nonuse, potentially because of failed viability criteria in damaged livers.

**Conclusions.:**

Short TTD was associated with inferior posttransplant outcome, challenging the dogma that shorter is better. Prolonged TTD did not negatively impact posttransplant outcomes, irrespective of NRP use. These findings support expanded use of liver donors with prolonged TTD, especially in the era of NRP and advanced perfusion, where viability assessment provides an additional safeguard.

## Graphical abstract

**Figure:**
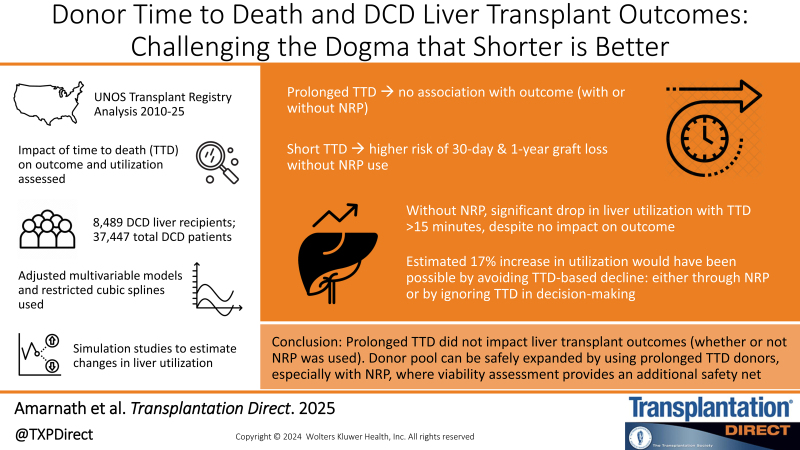

## INTRODUCTION

Liver transplantation is the standard of care for patients with end-stage liver disease. However, liver graft shortages continue to be a major challenge. Approximately 12% of patients in the US wait >5 y for a liver graft—many of whom either die or are removed from the waitlist because of deterioration in their clinical condition.^1^ Methods expanding the deceased donor pool and improving organ utilization from existing donors are required to prevent excess deaths on the waiting list.

Earlier studies demonstrated inferior outcomes for liver grafts procured from donors after circulatory death (DCD), which historically resulted in higher discard rates because of perceived risks of biliary complications and recipient morbidity when compared with donation after brainstem death (DBD) liver grafts.^2-4^ However, more recent evidence highlighted that although DCD grafts may have worse overall outcomes than DBD grafts, accepting a DCD liver graft offer often provides patients with a survival advantage when compared with waiting for a DBD offer.^5^ Additionally, the increasing use of novel perfusion technologies, such as normothermic regional perfusion (NRP), has shown real potential to further improve outcomes for DCD liver recipients.^6^

Following the withdrawal of life-sustaining treatment (WLST), donor warm ischemic time consists of time to death (TTD; withdrawal of treatment to asystole) followed by asystolic time (from asystole to abdominal aortic cold flush/NRP initiation). These are physiologically distinct time periods, with TTD having preservation of at least some blood flow, whereas asystolic time represents a period of absent blood flow. We have previously shown in other organs that these physiological differences do translate into a distinct impact of TTD and asystolic time on clinical outcomes.^7-9^ This highlights the importance of analyzing them separately rather than grouping them as donor warm ischemic time, as previous studies have done. TTD can vary significantly between donors because of fluctuations in hemodynamic parameters.^10^ Concerns persist that the potential ischemic insult from prolonged TTD may affect recipient outcomes. Some studies using UK data suggest that lengthy donor TTD may not be associated with worse graft outcomes.^7-9^ One study demonstrated that liver grafts from donors with short TTD may be linked to worse outcomes.^7^

In this study, we assessed the impact of donor TTD on liver graft outcomes and liver utilization in DCD transplantation, which has not been done previously.

## MATERIALS AND METHODS

### Setting

This population cohort study was performed using data collected from the United Network for Organ Sharing (UNOS) registry, specifically the standard transplant analysis and research and Donor Network supplement files. All data were provided in a fully anonymized format. Our Institutional Review Board equivalent determined that study-specific ethical review, approval, or informed consent were not required. We included adult recipients (aged 18 y and over at the time of transplant) of DCD liver-only transplants performed between January 1, 2010, and June 30, 2024. Exclusion criteria were multiorgan/multivisceral transplants, uncontrolled DCD and missing data for TTD and asystolic time. Data were extracted on June 30, 2025, which was the common closure date of the study, ensuring all patients had a minimum of 1-y posttransplant follow-up period.

### Outcomes and Definitions

The primary outcome was 1-y graft survival, which is defined in the UNOS registry as graft loss or death. Secondary outcomes were 1-y patient survival (mortality), early graft loss (defined as graft loss within 30 d), and length of stay (analyzed as time-to-discharge). Length of stay was censored at a maximum of 90 d for those still in hospital, or those who had died or lost their graft before 90 d.

TTD was defined as the time from WLST to donor asystole. Asystolic time was defined as the time from donor asystole to abdominal aorta cold flush. Functional TTD (FTTD) was the time from donor systolic blood pressure (SBP) falling <50 mm Hg until abdominal aorta cold flush.

### Cohorts for Analysis

To assess the impact of TTD on recipient outcomes, we included recipients of all livers recovered through super-rapid recovery (SRR) and NRP in the main cohort. Recipients of livers recovered using NRP were further analyzed as a separate cohort of specific interest, recognizing the rapid increase in use of this technique in recent years. As procurement technique (SRR or NRP) is not directly captured by UNOS, we used donor asystolic time (asystole to cold flush, which signifies the end of NRP in NRP cases) to define procurement technique. Donors >30 min from asystole to cold flush were defined as NRP; those with ≤30 min were classified as SRR. This approach to differentiate between SRR and NRP procurements has been used and validated previously.^11-13^

### Statistical Analysis

To account for missing data, multiple imputation was performed (aregImpute; Hmisc R package) to generate 20 imputed datasets.^14^ This approach for multiple imputation uses predictive mean matching with bootstraps to build rich additive restricted cubic spline models.^15,16^ This was chosen in preference to multiple imputation by chained equations, as it preserves nonlinear relationships. For survival outcomes (graft survival, patient survival, and length of stay), we included the event indicator variable and the cumulative hazard of the event in the multiple imputation model to preserve relationships between outcome and missing covariates.^17^ For utilization models, the variable indicating whether the liver was transplanted or not was included in the multiple imputation model for the same reason. Donors with missing data for TTD and asystolic time were excluded from the analysis, as these were our primary exposures of interest.

Multivariable Cox regression was performed to assess the impact of TTD on recipient graft survival, patient survival, and hospital length of stay. In addition, multivariable logistic regression was performed to assess the impact on early graft loss, and associations with utilization. Results were pooled from 20 imputed datasets, adjusting for variance based on both within- and between-imputation variation.^18^

Adjustment for a wide range of confounders was performed. Potential confounders were selected based on previous research and clinical expertise; statistical variable selection techniques (eg, stepwise selection) were avoided.^19^ We used hierarchical modeling to adjust for transplant center as a determinant of posttransplant outcome; this was done using a frailty Cox model allowing transplant center to be modeled as a random effect.

Restricted cubic splines with 4 knots (5th, 35th, 65th, and 95th percentiles) were used to analyze continuous variables known to have a strong correlation with the outcome or likely to have nonlinear relationships. An a priori decision was made to use splines for these variables to avoid assumptions of linearity. For continuous variables not modeled with splines, those with significant right skew on visual assessment of histograms were Log2-transformed. For such variables, the effect estimates therefore relate to the predictor doubling in value.

Additional models were built, which included interaction terms, as the impact of TTD on outcome may have differed with certain donor and transplant factors. Sensitivity analyses were also performed adjusting for additional potential confounders that were not included in the main models because of issues with missing data or multicollinearity.^20^

Kaplan-Meier plots were generated to show crude graft and patient survival, stratified by TTD occurrence. Continuous variables are given as median and interquartile range. Outputs of models are given as effect estimates with 95% confidence intervals (CIs). All analyses were performed in R (R Foundation for Statistical Computing, Vienna, Austria),^21^ using the following packages; tidyverse, rms, rmsMD, Hmisc, and survminer.^14-16,22-24^

### Simulation Studies Modeling

We performed an additional analysis to model how utilization rates would increase if either NRP was applied or organ decline based on TTD was avoided, following our previously described methodology.^25^ This modeling was performed in the SRR cohort as no inferior utilization was identified in the setting of NRP. Therefore, this stimulation study represents the potential number of additional liver transplants that would have been transplanted if either NRP were applied, or if TTD were ignored in donors following the SRR pathway. We identified that livers from donors with TTD of up to 10 min did not impact utilization. Based on this, within the cohort of donors with TTD of <10 min, we built a logistic regression model for utilization (as discussed above) excluding TTD as a factor. This model estimated the probability of a liver being used at the population level, accounting for all potential confounders (identified in previous modeling), within the cohort where TTD has been shown to not impact utilization.

We then applied this model to the cohort of donors with TTD of 15–30 min. This allowed us to predict the probability of acceptance if these livers were treated as if they had a TTD of <10 min by the same set of clinicians, while accounting for a wide range of donor factors influencing utilization.

Next, we compared the predicted utilization rates with the actual observed rates in the TTD of 15–30 min. The difference between the 2 estimates represents the additional number of livers that would have been transplanted if TTD was ignored in the decision-making process. To get a CI for this value, we used bootstrapping to construct simulated cohorts. Specifically, bootstrapping (with 10 000 bootstrapped samples) and percentile method were used to generate 95% CI using simulated cohorts to assess the uncertainty in the difference between predicted and actual utilization rates. We performed an identical analysis for donors with TTD of 30–45 min.

## RESULTS

Between January 1, 2010, and June 30, 2024, 8489 liver transplant recipients were included in the main cohort to analyze the impact of TTD on outcome, after excluding recipients with <1 y of follow-up. Of these, 703 recipients who received livers after NRP recovery were further analyzed as a separate cohort. To assess the impact of TTD on liver utilization, a total of 41 443 potential donors were included from January 1, 2010, to June 30, 2025 (follow-up was not required for utilization analyses), with 37 447 SRR and 3986 NRP patients.

**FIGURE 1.**
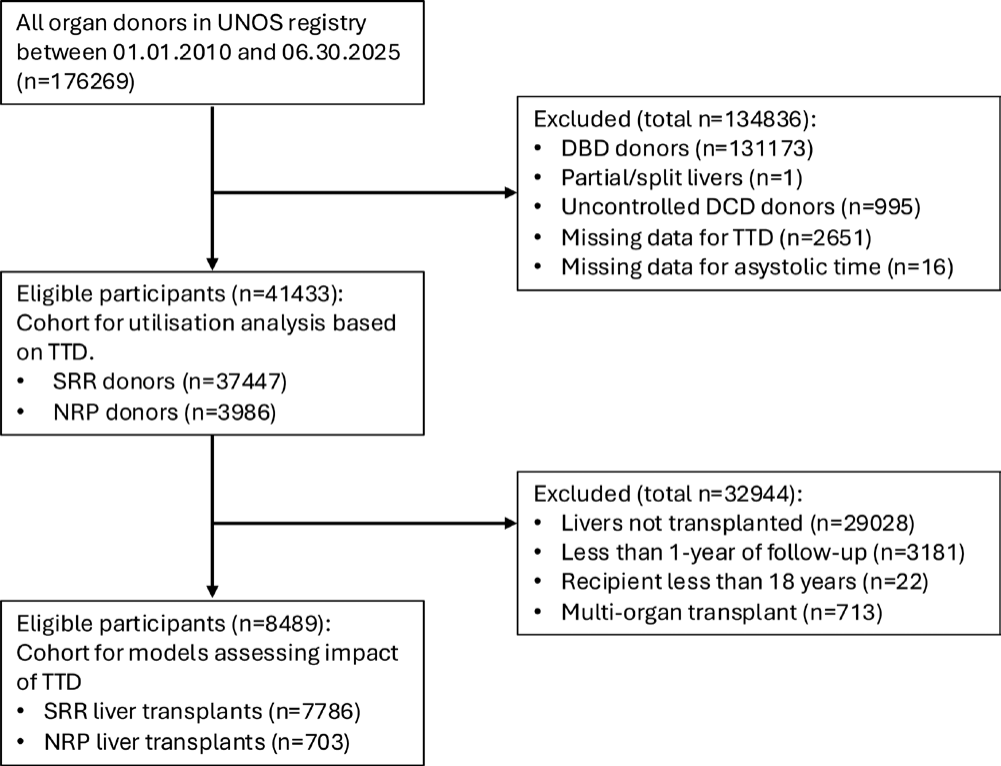
Flowchart and study cohorts. DBD, donation after brainstem death; DCD, donors after circulatory death; NRP, normothermic regional perfusion; SRR, super-rapid recovery; TTD, time to death; UNOS, United Network for Organ Sharing.

**FIGURE 2.**
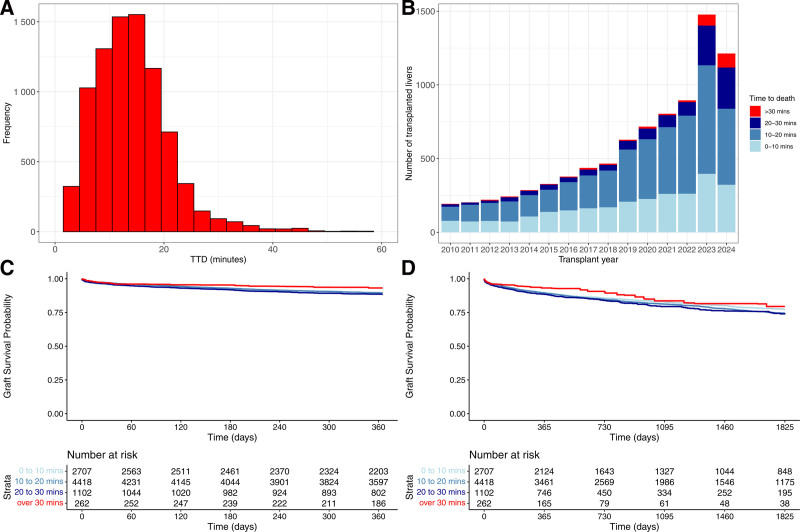
Cohort demographics and unadjusted survival. A, Total number of liver transplants based on TTD. B, Number of liver transplants over time based on TTD until June 30, 2024. C, Above—Kaplan-Meier curve of 1-y graft survival in the main cohort stratified by TTD. Below—Number at risk over the 1-y period. D, Above—Kaplan-Meier curve of 5-y graft survival in the main cohort stratified by TTD. Below—Number at risk over the 5-y period. TTD, time to death.

Information on cohort selection is given in our study flow diagram (Figure 1). Key cohort demographics are given in Table 1 (for the main cohort, with inclusion up to June 30, 2024) and **Table S1** (**SDC**, https://links.lww.com/TXD/A827, for the NRP cohort). Additional demographics and full description of missing data are in **Table S2** (**SDC**, https://links.lww.com/TXD/A827). The median TTD was 13 min (interquartile range, 9–17 min), with the distribution shown in Figure 2A and B. Crude 1- and 5-y graft survival data stratified by donor TTD are shown as Kaplan-Meier plots (Figure 2C and D).

**TABLE 1.** Main cohort demographics

Characteristics	Patients, N (%), N = 8489
Donor age, y	
Median (IQR)	39.0 (28.0–51.0)
Donor BMI	
Median (IQR)	26.7 (23.2–31.2)
Missing	78 (0.9%)
Donor sex	
Female	2808 (33.1%)
Male	5681 (66.9%)
Donor cause of death	
Cerebrovascular/stroke	1539 (18.1%)
Anoxia with PDCA	2438 (28.7%)
Drug overdose	1218 (14.3%)
Head trauma	2183 (25.7%)
Other	804 (9.5%)
Missing	307 (3.6%)
Time to death, min	
Median (IQR)	13.0 (9.00–17.0)
NRP status	
SRR	7786 (91.7%)
NRP	703 (8.3%)
Recipient age, y	
Median (IQR)	59.0 (51.0–64.0)
Recipient sex	
Female	2775 (32.7%)
Male	5714 (67.3%)
Ex situ machine perfusion	
None	5436 (64.0%)
Normothermic	1733 (20.4%)
Hypothermic	84 (1.0%)
Other	66 (0.8%)
Missing	1170 (13.8%)
Cold ischemic time, h	
Median (IQR)	6.00 (4.70–9.40)
Missing	63 (0.7%)
Donor peak ALT	
Median (IQR)	82.0 (39.0–189)
Missing	11 (0.1%)

ALT, alanine aminotransferase; BMI, body mass index; IQR, interquartile range; NRP, normothermic regional perfusion; PDCA, pre-donation cardiac arrest; SRR, super-rapid recovery.

### Impact of Donor TTD on Recipient Outcomes

**FIGURE 3.**
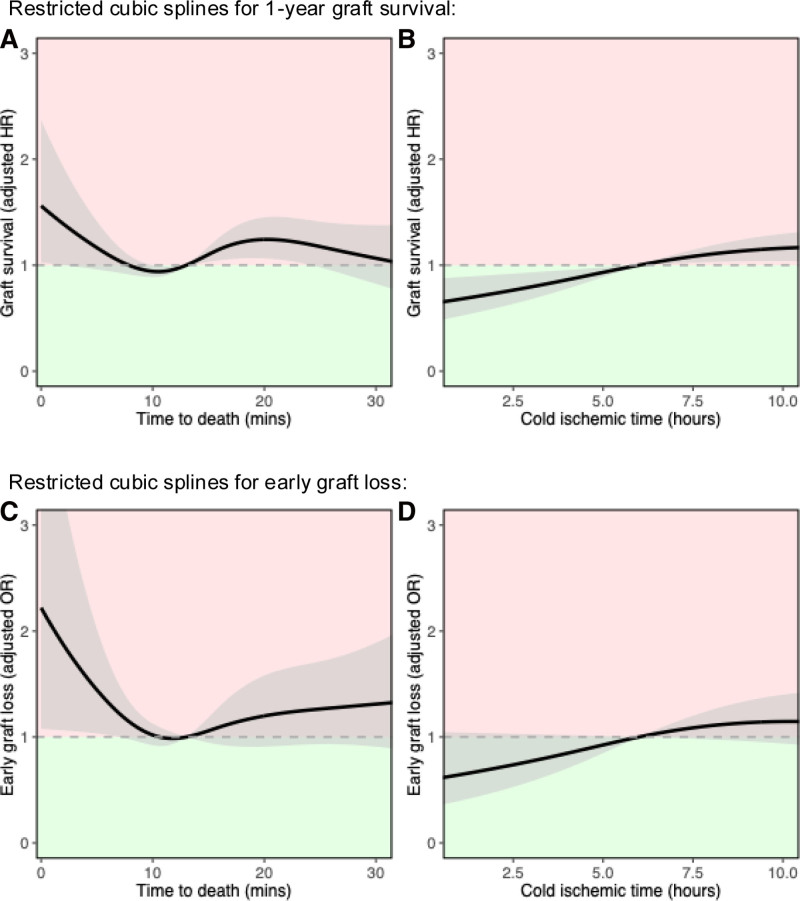
Impact on 1-y graft survival and early graft loss using restricted cubic splines with 4 knots in the main cohort. Lines represent restricted cubic splines with gray shaded areas for 95% confidence intervals. Associations between TTD (A) and cold ischemic time (B) against 1-y graft survival (derived from model in Table 2). Associations between TTD (C) and cold ischemic time (D) against early graft loss (derived from model in **Table S3** [**SDC**, https://links.lww.com/TXD/A827]). The green region represents superior outcome compared with the reference, while the red region signifies inferior outcome. HR, hazard ratio; OR, odds ratio; TTD, time to death.

A multivariable Cox regression model was used to assess the association of TTD with recipient 1-y graft survival in the main cohort, adjusting for a wide range of factors (Table 2; splines in Figure 3A and B; **Figure S1** [**SDC**, https://links.lww.com/TXD/A827]). Donor TTD demonstrated a significant nonlinear association with graft survival (spline *P* = 0.032). As seen in Figure 3A, short TTD was associated with worse graft survival, while prolonged TTD was not linked with worse outcome.

**TABLE 2.** Multivariable Cox model for 1-y graft survival in the main cohort, pooled from 20 imputed datasets

Variable	HR (95% CI)	*P*
^*a*^RCS term: time to death	Wald test	0.032
NRP status		
SRR	Ref	–
NRP	0.528 (0.372-0.749)	<0.001
Donor BMI (per 5 units)	1.009 (0.951-1.070)	0.774
Donor sex		
Female	Ref	–
Male	1.164 (1.002-1.353)	0.048
Donor hypertension history		
No	Ref	–
Yes	1.307 (1.107-1.542)	0.002
Donor cause of death		
Cerebrovascular/stroke	Ref	–
Anoxia with PDCA	0.691 (0.561-0.850)	<0.001
Drug overdose	0.614 (0.470-0.802)	<0.001
Head trauma	0.755 (0.612-0.932)	0.009
Other	0.597 (0.447-0.797)	<0.001
Static cold storage solution		
UW	Ref	–
HTK	1.049 (0.885-1.243)	0.583
Other	0.943 (0.782-1.136)	0.536
Ex situ machine perfusion		
None	Ref	–
Normothermic	0.544 (0.412-0.718)	<0.001
Hypothermic	0.167 (0.044-0.636)	0.009
Unspecified	0.506 (0.187-1.368)	0.179
Recipient age (per 10 y)	1.066 (0.990-1.149)	0.090
Recipient BMI (per 5 units)	1.037 (0.975-1.102)	0.251
Recipient sex		
Female	Ref	–
Male	1.197 (1.026-1.397)	0.022
MELD score (per 10 units)	1.126 (1.010-1.256)	0.033
Recipient dialysis		
No	Ref	–
Yes	1.449 (1.018-2.063)	0.039
Recipient diabetes		
No	Ref	–
Yes	1.259 (1.086-1.461)	0.002
Status 1A		
No	Ref	–
Yes	3.046 (1.700-5.459)	<0.001
Recipient primary diagnosis		
Alcoholic liver disease	Ref	–
HCC	1.249 (1.004-1.553)	0.045
NASH	1.303 (1.039-1.634)	0.022
Cholestatic disease	1.199 (0.859-1.674)	0.286
Acute liver failure	0.983 (0.518-1.864)	0.958
HCV	1.277 (0.964-1.691)	0.089
Other/unknown	1.474 (1.177-1.846)	<0.001
Recipient medical condition at TX		
Not hospitalized	Ref	–
Hospitalized, but not in ICU	1.260 (1.015-1.565)	0.036
In ICU	2.188 (1.557-3.073)	<0.001
Recipient functional status (higher = better status)		
≤60%	Ref	–
70%	1.199 (1.012-1.420)	0.036
80%	0.946 (0.765-1.171)	0.610
90%	0.756 (0.503-1.136)	0.178
100%	0.587 (0.261-1.320)	0.198
Recipient ethnicity		
White	Ref	–
Asian	0.811 (0.531-1.240)	0.334
Black	1.247 (0.948-1.640)	0.114
Hispanic	0.897 (0.736-1.094)	0.283
Other	1.635 (1.091-2.450)	0.017
Log2-days on liver waitlist	1.056 (1.021-1.092)	0.001
^*a*^RCS term: donor age	Wald test	0.863
^*a*^RCS term: donor peak ALT	Wald test	0.666
^*a*^RCS term: transplant year	Wald test	0.015
^*a*^RCS term: cold ischemic time	Wald test	0.018
^*a*^RCS term: admission to donation time	Wald test	<0.001

Right-skewed variables not modeled as splines were log2-transformed, so the results relate to change every time the variable doubles. ‟–” represents the reference category, for which no *P* value is applicable.

^*a*^For restricted cubic splines, see Figure 3A and B; **Figure S1** (**SDC**, https://links.lww.com/TXD/A827).

ALT, alanine aminotransferase; BMI, body mass index; CI, confidence interval; HCC, hepatocellular carcinoma; HCV, hepatitis C virus; HR, hazard ratio; HTK, histidine-tryptophan-ketoglutarate; ICU, intensive care unit; MELD, model for end-stage liver disease; NASH, nonalcoholic steatohepatitis; NRP, normothermic regional perfusion; PDCA, pre-donation cardiac arrest; RCS, restricted cubic spline; Ref, reference; SRR, super-rapid recovery; TX, transplantation; UW, University of Wisconsin.

Similar trends of worse outcome with short TTD was observed for early graft loss (spline *P* = 0.149; **Table S3** [**SDC**, https://links.lww.com/TXD/A827]; splines in Figure 3C and D; **Figure S2** [**SDC**, https://links.lww.com/TXD/A827]), 1-y patient survival (spline *P* = 0.125; **Table S4** [**SDC**, https://links.lww.com/TXD/A827]; splines in **Figure S3** [**SDC**, https://links.lww.com/TXD/A827]), and recipient hospital length of stay (spline *P* = 0.027; **Table S5** [**SDC**, https://links.lww.com/TXD/A827]; splines in **Figure S4** [**SDC**, https://links.lww.com/TXD/A827]).

We built an additional saturated model including the following additional confounders: recipient encephalopathy grade, donor serum sodium, macrosteatosis percentage on biopsy, donor peak creatinine, donor peak bilirubin, allocation type, donor peak international normalized ratio, and donor peak serum sodium. These variables did not change the conclusions of the model.

To adjust for potential individual transplant center effects, a Cox frailty model for 1-y graft survival with random effects for transplant center was performed, which did not meaningfully change our conclusions.

Additional multivariable Cox regression models were built to analyze the impact of TTD on longer-term 5-y graft and patient survival, adjusting for the same factors in Table 2. Prolonged TTD was again not associated with longer-term outcomes (**Figures S5** and **S6**, **SDC**, https://links.lww.com/TXD/A827).

Some suggest that FTTD is a better metric for indicating the level of ischemic injury before death. We applied the same modeling strategy as described above to analyze the impact of FTTD on 1-y graft survival. Donor FTTD defined as SBP <50 mm Hg was not significantly associated with graft survival (spline *P* = 0.896; **Table S6** [**SDC**, https://links.lww.com/TXD/A827]; splines in **Figure S7** [**SDC**, https://links.lww.com/TXD/A827]).

We performed further exploratory analyses to assess whether short TTD donors were more unstable, and we found no evidence that short TTD was associated with cardiovascular support, respiratory support, or hemodynamic observations (**Table S7**, **SDC**, https://links.lww.com/TXD/A827).

To analyze the impact of asystolic time on outcome, we built models similar to those for TTD, but now also including asystolic time in the models in the SRR cohort (asystolic time was not available for NRP donors). Increasing asystolic time showed trends toward worse outcome for 1-y graft survival (spline *P* = 0.426; **Figure S8A** [**SDC**, https://links.lww.com/TXD/A827]) and 1-y patient survival (spline *P* = 0.908; **Figure S8B** [**SDC**, https://links.lww.com/TXD/A827]), although not statistically significant. However, significant association between prolonged asystolic time and worse outcome was identified for 5-y graft survival (spline *P* = 0.018; **Figure S8C** [**SDC**, https://links.lww.com/TXD/A827]) and 5-y patient survival (spline *P* = 0.043; **Figure S8D** [**SDC**, https://links.lww.com/TXD/A827]). The lack of statistical significance at 1 y may be attributed to small effective sample size (number of events).

### NRP Cohort

A limited multivariable analysis was performed to assess the impact of TTD on 1-y graft survival in the setting of NRP, adjusting for machine perfusion, cold ischemic time, and donor age. No association was observed between donor TTD and graft survival (spline *P* = 0.931; see splines in **Figure S9A**, **SDC**, https://links.lww.com/TXD/A827). The limited number of events (graft losses) prevented more multivariable analyses with a greater number of confounders.

Similarly, TTD was not associated with 30-d graft loss (spline *P* = 0.844), 1-y patient survival (spline *P* = 0.904), and hospital LOS (spline *P* = 0.524; see splines in **Figure S9B–D**, **SDC**, https://links.lww.com/TXD/A827). Longer-term 5-y graft (spline *P* = 0.758) and 5-y patient survival (*P* = 0.835) were also not associated with donor TTD.

We then applied the same modeling strategy as described above to analyze the impact of FTTD on graft survival with NRP use. Donor FTTD defined as SBP <50 mm Hg was not associated with graft survival (spline *P* = 0.235).

### Impact of Donor TTD on Utilization

After demonstrating that prolonged TTD was not associated with inferior posttransplant outcome in adjusted analyses, we sought to assess whether prolonged TTD impacted utilization. A multivariable logistic model was built to analyze the impact of donor TTD on liver utilization in the cohort of all DCD donors, whether transplanted or not (n = 41 433). As with the models for recipient outcomes, we modeled TTD as a spline to capture its nonlinear relationship with utilization. Donor TTD was significantly associated with liver utilization (spline *P* < 0.001). Interaction terms demonstrated significant differences in the impact of TTD on utilization between SRR and NRP donors (interaction *P* < 0.001).

**FIGURE 4.**
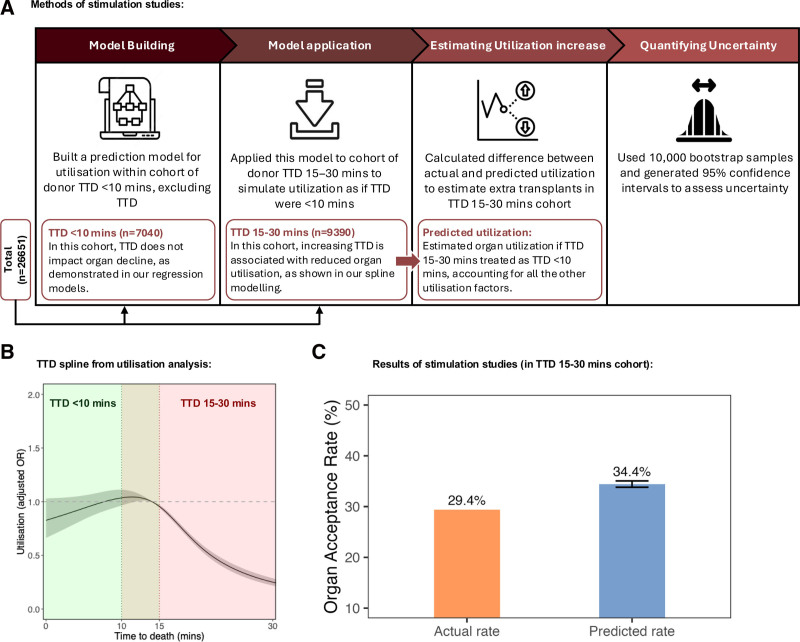
Assessing the impact of TTD on utilization, and potential extra transplants if TTD-based decline was avoided. A, Descriptive graphic illustrating the methods of simulation studies. B, Association between TTD against utilization in the super-rapid recovery (SRR) cohort utilizing restricted cubic splines with 4 knots. Lines represent restricted cubic splines with gray shaded area for 95% confidence intervals. The green region represents the TTD <10 min cohort with greater utilization. The red region shows the TTD 15–30 min cohort where a decrease in utilization is observed. C, Results of the stimulation studies in the cohort of donors with TTD of 15–30 min. OR, odds ratio; TTD, time to death.

In the SRR cohort (n = 37 447), utilization began to decline rapidly at donor TTD of >10–15 min (Figure 4B; Table 3; splines in **Figure S10** [**SDC**, https://links.lww.com/TXD/A827]). Given that this analysis was adjusted for other donor factors, and we have shown that TTD >10–15 min did not negatively impact outcomes, this represents a large number of missed opportunities for organ utilization.

**TABLE 3.** Multivariable logistic model for utilization in the SRR cohort, pooled from 20 imputed datasets

Variable	OR (95% CI)	*P*
^*a*^RCS term: time to death	Wald test	<0.001
Donor blood group		
A	Ref	–
AB	0.236 (0.190-0.294)	<0.001
B	0.717 (0.648-0.793)	<0.001
O	1.022 (0.961-1.087)	0.483
Donor BMI (per 5 units)	0.800 (0.781-0.820)	<0.001
Donor sex		
Female	Ref	–
Male	1.023 (0.961-1.089)	0.481
Donor hypertension history		
No	Ref	–
Yes	0.812 (0.755-0.873)	<0.001
Donor diabetes history		
No	Ref	–
Yes	0.902 (0.815-0.997)	0.043
Donor cause of death		
Cerebrovascular/stroke	Ref	–
Anoxia with PDCA	1.181 (1.072-1.301)	<0.001
Drug overdose	1.173 (1.042-1.321)	0.008
Head trauma	1.033 (0.938-1.138)	0.510
Other	0.933 (0.830-1.048)	0.244
Ex situ machine perfusion		
None	Ref	–
Normothermic	68.996 (58.721-81.068)	<0.001
Hypothermic	49.398 (27.369-89.156)	<0.001
Unspecified	13.843 (7.966-24.059)	<0.001
Donor peak serum sodium	1.014 (1.011-1.018)	<0.001
^*a*^RCS term: asystolic time	Wald test	<0.001
^*a*^RCS term: donor age	Wald test	<0.001
^*a*^RCS term: donor peak ALT	Wald test	<0.001
^*a*^RCS term: donor peak bilirubin	Wald test	<0.001
^*a*^RCS term: peak albumin	Wald test	<0.001
^*a*^RCS term: year of donation	Wald test	<0.001

Right-skewed variables not modeled with splines were log2-transformed, so the results relate to change every time the variable doubles. ‟–” represents the reference category, for which no *P* value is applicable.

^*a*^For restricted cubic splines, see Figure 4B; **Figure S10** (**SDC**, https://links.lww.com/TXD/A827).

ALT, alanine aminotransferase; BMI, body mass index; CI, confidence interval; OR, odds ratio; PDCA, pre-donation cardiac arrest; RCS, restricted cubic spline; Ref, reference; SRR, super-rapid recovery.

In the NRP cohort (n = 3986), donor TTD was a significant factor for utilization (spline *P* = 0.002; **Table S8** [**SDC**, https://links.lww.com/TXD/A827]; splines in **Figure S11** [**SDC**, https://links.lww.com/TXD/A827]); however, the nature of the relationship was very different. In the NRP cohort, there was a decrease in the utilization of livers from donors with short TTD, which contrasted with the trend observed in the SRR cohort. We speculate that this represents short TTD livers failing NRP viability criteria.

### Simulation Studies for Predicted Increase in Utilization

We have demonstrated that prolonged TTD does not impact liver transplant outcomes. Therefore, declining a liver based on TTD 15–30 min represents an inappropriate organ decline; avoiding such declines could allow for safe transplantation of more livers. These declines occurred only among SRR donors, with no decreased utilization seen with NRP. To estimate the increase in liver utilization if either NRP was used or TTD was ignored in the decision-making among SRR donors, we used a methodology previously developed by our group,^25^ which is illustrated in the descriptive graphic shown in Figure 4.

The actual acceptance rate of livers from donors with a TTD of 15–30 min in the SRR cohort was 29.4%. In this cohort, the predicted acceptance rate, had clinicians treated these organ offers identically to offers from donors with TTD<10 min, was 34.4% (95% CI, 33.8%-35.1%), adjusting for all factors in Table 3 except TTD. This corresponds to a relative increase in utilization of 17.1% (95% CI, 15.0%-19.3%). An estimated 654 additional livers (95% CI, 572-735) from donors with TTD 15–30 min could have been transplanted over the study period (Table 4).

**TABLE 4.** Modeling impact of avoiding TTD-based liver decline in the SRR cohort

Outcome measure	Donor TTD 15–30 min	Donor TTD of 30–45 min
Actual acceptance rate (2010–2024)	29.4%	12.1%
Predicted acceptance rate (95% CI)	34.4% (33.8%-35.1%)	30.6% (29.7%-31.5%)
Absolute increase in acceptance (95% CI)	5.0% (4.4%-5.7%)	18.5% (17.5%-19.4%)
Relative increase in acceptance (95% CI)	17.1% (15.0%-19.3%)	152.7% (144.9-160.4%)
Extra livers (95% CI)	654 (573-736)	547 (519-574)

Predicted acceptance rate is the predicted rate of acceptance if these livers were treated identically to livers from donors with a TTD 0–10 min (ie, if TTD did not influence decision to decline). Absolute and relative increase rates were calculated. CIs were generated using bootstrapping.

CI, confidence interval; SRR, super-rapid recovery; TTD, time to death.

Additionally, we estimated the potential increase in liver utilization for donors with TTD of 30–45 min. Since the safety of liver transplantation from this cohort is less certain because of limited data, we analyzed this cohort separately. The actual acceptance rate was 12.1%, whereas predicted acceptance rate was 30.6% (95% CI, 29.7%-31.5%), if these offers were treated identically to offers with TTD <10 min. This reflects a relative increase of 152.7% (95% CI, 144.9%-160.4%). This represents an estimated 547 extra livers (95% CI, 519-574) over the study period (Table 4). This cohort of donors with TTD of 30–45 min would be especially suitable for NRP, which could enable their safe use in transplantation.

## DISCUSSION

This is the largest study to date analyzing the impact of TTD on liver transplant outcomes, and the first to examine its effect on liver utilization. Prolonged TTD did not show inferior posttransplant outcomes, irrespective of whether NRP was used. We uniquely simulated the potential increase in organ transplants if declines based on TTD were avoided among SRR donors. These simulations demonstrated significant increases in liver transplant utilization could be achieved by avoiding TTD-based decline in setting of SRR, or by using NRP in this donor cohort. The exponential increase in the use of DCD donors in the United States, as well as the rising use of NRP, highlights the importance of these findings.^26^

We demonstrated no significant relationship between increased TTD and graft survival after DCD liver transplantation, suggesting that liver grafts are relatively protected from ischemic injury during the time interval between WLST and cardiac asystole. However, livers from donors with short TTD had worse graft survival. Our primary hypothesis is that donors with short TTD have incurred severe brainstem injury but not become DBD donors, perhaps because of technical reasons preventing declaration of brain death or progression to brain death after initial testing. Severe brainstem injury leads to respiratory center failure,^27^ resulting in rapid death and near-instant TTD. Additionally, these donors suffer from a combination of both significant pro-inflammatory cytokine storm triggered by severe brainstem injury,^28^ as well as warm ischemia-reperfusion injury inherent in the DCD pathway.^29^ We hypothesize that this dual insult makes these livers particularly susceptible to adverse outcomes.

We hypothesize that the liver injury seen with short TTD manifests differently in the SRR and NRP cohort. In the SRR cohort, this injury results in inferior posttransplant outcome. In contrast, NRP allows for viability assessment, where liver injury may lead to reduced utilization (because of failing viability criteria) rather than inferior posttransplant outcome. The differences in the impact of TTD on posttransplant outcomes and utilization between NRP and SRR cohorts reinforces that association of short TTD with increased liver injury.

Similar findings of prolonged TTD not being a risk factor and short TTD being associated with worse outcomes have been reported in a previous study.^7^ Our study builds on this by analyzing a large sample size and providing external validation in a different transplantation setting. We also analyzed the impact of TTD on liver utilization and simulated the increase in liver transplants if TTD-based decision-making was ignored. Furthermore, our analysis included a larger cohort of NRP donors, especially from recent years in the utilization models, allowing us to analyze the trends seen with donor TTD.

The increasing use of ex situ normothermic machine perfusion and NRP in the United States has significantly expanded the donor pool.^30^ These technologies provide a safety net by enabling viability assessment of donor livers, which can help further push boundaries of stand-down times while reducing any associated risks.^31^ By improving the functional assessment of livers before transplantation, normothermic machine perfusion and NRP can further optimize liver utilization, potentially reducing organ waste and improving overall transplant outcomes.^32,33^ Therefore, NRP and other new technologies should be more aggressively used in this cohort of donors with prolonged TTD, making our findings highly relevant in the current landscape of NRP use.

We avoided using the stated “reason for discard” for the utilization analysis as decisions to discard are almost always multifactorial and rarely attributable to a single reason. For example, a liver may be declined because of donor age in the context of prolonged TTD, which otherwise may have been accepted if TTD was shorter. In this setting, attributing the discard solely to donor age would be misleading. To overcome this issue, we focused on modeling the actual contributing factors and outcome of the decision.

Much work was done to extract and clean the data by pulling data from both the UNOS standard transplant analysis and research and Donor Network supplement files, and the majority of donor timing data was accessible. However, there are a few limitations associated with this study. The main limitation is the retrospective design and potential for selection bias. However, we adjusted for a wide range of potential confounders, which was possible because of the large cohort size. Additionally, similar patterns observed in the UK data suggest selection bias is unlikely to explain our findings. Another limitation is the inevitable degree of missing data, but robust techniques were used to impute this data. Because of lack of granularity and inherited constraints in the cardiorespiratory support data, analyses related to donor instability were kept purely as exploratory.

To conclude, short TTD was associated with inferior outcomes, potentially because of severe brainstem injury and cytokine storm compounding subsequent ischemia. In contrast, prolonged TTD did not negatively impact outcomes, indicating that TTD should not be used to make donor selection. Therefore, livers from donors with TTD of >15 min are being underutilized despite good potential for transplantation. These livers could have been safely transplanted by avoiding TTD-based declines or with the use of NRP. This cohort of donors with prolonged TTD should be prioritized by organ procurement organizations to actively use NRP and other advanced perfusion techniques to safely extend the stand-down times and maximize organ recovery, with viability assessment providing an additional safeguard.

## ACKNOWLEDGMENTS

The authors thank the organ donors, their families, donor coordinators, and the wider transplant team, without whom this research would not be possible. Also, the authors thank transplant centers for collecting data and the statistics team for providing data from the UNOS Transplant Registry, as well as their time and effort in supporting the development of this study.

## Supplementary Material


