# Supplementary material for: Donor Time to Death and DCD Liver Transplant Outcomes: Challenging the Dogma That Shorter Is Better
Source: Transplant Direct. 2026 Feb 11;12(3):e1911. doi: 10.1097/TXD.0000000000001911 (PMC12900224; doi:10.1097/TXD.0000000000001911)
Supplement: Supplementary file 1 [file txd-12-e1911-s001.pdf]

Supplementary Digital Content (SDC)

Table S1: NRP cohort demographics. Abbreviations: BMI, body mass index; PDCA, pre-donation cardiac arrest; TTD, time to death; UW, University of Wisconsin; HTK, histidine-tryptophan-ketoglutarate; HBV, hepatitis B virus; ICU, Intensive care unit; MELD, model for end-stage liver disease; HCC, hepatocellular carcinoma; NASH, nonalcoholic steatohepatitis; HCV, hepatitis C virus; ALT, alanine aminotransferase; INR, international normalized ratio; PTT, partial thromboplastin time.

Characteristics	Overall (N=703)
**Donor age (years)**	
Median [IQR]	42.0 [30.0,55.0]
**Donor BMI**	
Median [IQR]	27.2 [23.6,32.0]
Missing	9 (1.3%)
**Donor sex**	
Female	210 (29.9%)
Male	493 (70.1%)
**Donor cause of death**	
Cerebrovascular/stroke	118 (16.8%)
Anoxia with PDCA	193 (27.5%)
Drug overdose	105 (14.9%)
Head Trauma	194 (27.6%)
Other	62 (8.8%)
Missing	31 (4.4%)
**Time to death (mins)**	
Median [IQR]	15.0 [11.0,18.0]
**Functional TTD (from 50 mmHg)**	
Median [IQR]	2.00 [0,5.00]
**Functional TTD (from 80 mmHg)**	
Median [IQR]	5.00 [2.00,11.0]
**Donor admission-to-donation time (days)**	
Median [IQR]	6.00 [4.00,9.00]
Missing	3 (0.4%)
**Static cold storage solution**	
UW	352 (50.1%)
HTK	91 (12.9%)
Other	260 (37.0%)
**MELD score**	
Median [IQR]	19.0 [14.0,24.0]
**Recipient serum albumin**	

Median [IQR]	3.20 [2.80,3.60]
Missing	1 (0.1%)
**Recipient encephalopathy**	
None	255 (36.3%)
Grade 1-2	381 (54.2%)
Grade 3-4	67 (9.5%)
**Recipient ascites**	
Absent	151 (21.5%)
Slight	368 (52.3%)
Moderate	184 (26.2%)
**Recipient dialysis status**	
No	688 (97.9%)
Yes	12 (1.7%)
Missing	3 (0.4%)
**Recipient age (years)**	
Median [IQR]	59.0 [51.0,65.0]
**Recipient BMI**	
Median [IQR]	28.4 [25.3,32.7]
Missing	1 (0.1%)
**Recipient sex**	
Female	216 (30.7%)
Male	487 (69.3%)
**Treated for rejection within 1 year**	
No	443 (63.0%)
Yes	52 (7.4%)
Missing	208 (29.6%)
**Treated for rejection within 6 months**	
No	541 (77.0%)
Yes	46 (6.5%)
Missing	116 (16.5%)
**Recipient medical condition at listing**	
Not Hospitalised	600 (85.3%)
Hospitalised, but not in ICU	87 (12.4%)
In ICU	16 (2.3%)
**Allocation type**	
Local	445 (63.3%)
Regional	131 (18.6%)
National	127 (18.1%)
**Donor diabetes**	
No	600 (85.3%)
Yes	99 (14.1%)
Missing	4 (0.6%)
**Recipient diabetes**	

No	494 (70.3%)
Yes	209 (29.7%)
**Donor hypertension**	
No	459 (65.3%)
Yes	237 (33.7%)
Missing	7 (1.0%)
**Donor peak serum sodium**	
Median [IQR]	150 [146,157]
**Ex-situ machine perfusion**	
None	522 (74.3%)
Normothermic	171 (24.3%)
Hypothermic	4 (0.6%)
Other	2 (0.3%)
Missing	4 (0.6%)
**Super-Urgent Candidate Status**	
No	701 (99.7%)
Yes	2 (0.3%)
**Recipient Primary Diagnosis at Listing**	
Alcoholic Liver Disease	221 (31.4%)
HCC	154 (21.9%)
NASH	141 (20.1%)
Cholestatic Disease	51 (7.3%)
Acute Liver Failure	5 (0.7%)
HCV	19 (2.7%)
Others	105 (14.9%)
Missing	7 (1.0%)
**Graft loss within 30 days**	
No	691 (98.3%)
Yes	12 (1.7%)
**Recipient Functional Status Percentage**	
≤60%	361 (51.4%)
70%	195 (27.7%)
80%	111 (15.8%)
90%	24 (3.4%)
100%	5 (0.7%)
Missing	7 (1.0%)
**Donor ethnicity**	
White, Non-Hispanic	548 (78.0%)
Black, Non-Hispanic	6 (0.9%)
Hispanic/Latino	58 (8.3%)
Other	79 (11.2%)
**Recipient ethnicity**	

White, Non-Hispanic	547 (77.8%)
Black, Non-Hispanic	13 (1.8%)
Hispanic/Latino	24 (3.4%)
Other	94 (13.4%)
**Liver waitlist duration**	
Median [IQR]	69.0 [19.0,215]
**Macrosteatosis fat percentage on biopsy**	
Median [IQR]	5.00 [0,10.0]
Missing	422 (60.0%)
**Microsteatosis fat percentage on biopsy**	
Median [IQR]	0 [0,5.00]
Missing	433 (61.6%)
**Cold ischemic time (hours)**	
Median [IQR]	5.37 [4.07,8.99]
Missing	4 (0.6%)
**Donor peak AST**	
Median [IQR]	112 [60.0,285]
Missing	1 (0.1%)
**Donor peak ALT**	
Median [IQR]	82.0 [40.3,194]
Missing	1 (0.1%)
**Donor peak creatinine (mg/dL)**	
Median [IQR]	1.30 [1.00,1.89]
Missing	1 (0.1%)
**Donor peak bilirubin**	
Median [IQR]	0.800 [0.600,1.20]
Missing	1 (0.1%)

Table S2: Full main cohort demographics. TTD = time to death; BMI = Body mass index; PDCA = pre-donation cardiac arrest; UW = University of Wisconsin; HTK = histidine-tryptophan-ketoglutarate; TX = Transplantation; MELD = Model for end-stage liver disease; HCC: Hepatocellular carcinoma; NASH = Nonalcoholic steatohepatitis; HCV = Hepatitis C virus; ICU = Intensive care unit; ALT = Alanine aminotransferase.

Characteristics	Patients, No. (%) (N=8489)
**Donor age (years)**	
Median [IQR]	39.0 [28.0,51.0]
**Donor BMI**	
Median [IQR]	26.7 [23.2,31.2]
Missing	78 (0.9%)
**Donor sex**	
Female	2808 (33.1%)
Male	5681 (66.9%)
**Donor cause of death**	
Cerebrovascular/stroke	1539 (18.1%)
Anoxia with PDCA	2438 (28.7%)
Drug overdose	1218 (14.3%)
Head Trauma	2183 (25.7%)
Other	804 (9.5%)
Missing	307 (3.6%)
**Time to death (mins)**	
Median [IQR]	13.0 [9.00,17.0]
**NRP status**	
SRR	7786 (91.7%)
NRP	703 (8.3%)
**Functional TTD (from 50 mmHg)**	
Median [IQR]	2.00 [0,4.00]
**Functional TTD (from 80 mmHg)**	
Median [IQR]	5.00 [2.00,9.00]
**Donor admission-to-donation time (days)**	
Median [IQR]	5.00 [3.00,8.00]
Missing	34 (0.4%)
**Static cold storage solution**	
UW	3773 (44.4%)
HTK	1773 (20.9%)
Other	2919 (34.4%)
Missing	24 (0.3%)
**MELD score**	
Median [IQR]	18.0 [13.0,24.0]

Recipient serum albumin	
Median [IQR]	3.20 [2.80,3.60]
Missing	9 (0.1%)
**Recipient encephalopathy**	
None	3113 (36.7%)
Grade 1-2	4600 (54.2%)
Grade 3-4	774 (9.1%)
Missing	2 (0.0%)
**Recipient ascites**	
Absent	2122 (25.0%)
Slight	4036 (47.5%)
Moderate	2329 (27.4%)
Missing	2 (0.0%)
**Recipient dialysis status**	
No	8218 (96.8%)
Yes	227 (2.7%)
Missing	44 (0.5%)
**Recipient age (years)**	
Median [IQR]	59.0 [51.0,64.0]
**Recipient BMI**	
Median [IQR]	28.4 [24.9,32.6]
Missing	15 (0.2%)
**Recipient sex**	
Female	2775 (32.7%)
Male	5714 (67.3%)
**Treated for rejection within 1 year**	
No	5580 (65.7%)
Yes	678 (8.0%)
Missing	2231 (26.3%)
**Treated for rejection within 6 months**	
No	6116 (72.0%)
Yes	613 (7.2%)
Missing	1760 (20.7%)
**Recipient medical condition at listing**	
Not Hospitalised	7213 (85.0%)
Hospitalised, but not in ICU	971 (11.4%)
In ICU	305 (3.6%)
**Allocation type**	
Local	4851 (57.1%)
Regional	2202 (25.9%)
National	1436 (16.9%)
**Donor diabetes**	
No	7564 (89.1%)

Yes	873 (10.3%)
Missing	52 (0.6%)
**Recipient diabetes**	
No	5871 (69.2%)
Yes	2610 (30.7%)
Missing	8 (0.1%)
**Donor hypertension**	
No	5888 (69.4%)
Yes	2535 (29.9%)
Missing	66 (0.8%)
**Donor peak serum sodium**	
Median [IQR]	151 [145,158]
Missing	13 (0.2%)
**Ex-situ machine perfusion**	
None	5436 (64.0%)
Normothermic	1733 (20.4%)
Hypothermic	84 (1.0%)
Other	66 (0.8%)
Missing	1170 (13.8%)
**Super-Urgent Candidate Status**	
No	8430 (99.3%)
Yes	59 (0.7%)
**Recipient Primary Diagnosis at Listing**	
Alcoholic Liver Disease	2401 (28.3%)
HCC	2141 (25.2%)
NASH	1498 (17.6%)
Cholestatic Disease	510 (6.0%)
Acute Liver Failure	88 (1.0%)
HCV	655 (7.7%)
Others/Unknown	1133 (13.3%)
Missing	63 (0.7%)
**Graft loss within 30 days**	
No	8191 (96.5%)
Yes	294 (3.5%)
Missing	4 (0.0%)
**Recipient Functional Status Percentage**	
≤60%	4809 (56.6%)
70%	1879 (22.1%)
80%	1287 (15.2%)
90%	339 (4.0%)
100%	90 (1.1%)
Missing	85 (1.0%)

**Donor ethnicity**	
White, Non-Hispanic	6507 (76.7%)
Black, Non-Hispanic	149 (1.8%)
Hispanic/Latino	898 (10.6%)
Other	827 (9.7%)
**Recipient ethnicity**	
White, Non-Hispanic	6337 (74.6%)
Black, Non-Hispanic	272 (3.2%)
Hispanic/Latino	447 (5.3%)
Other	1246 (14.7%)
**Liver waitlist duration**	
Median [IQR]	97.0 [24.0,254]
Missing	22 (0.3%)
**Macrosteatosis fat percentage on biopsy**	
Median [IQR]	5.00 [0,10.0]
Missing	5984 (70.5%)
**Microsteatosis fat percentage on biopsy**	
Median [IQR]	0 [0,5.00]
Missing	6081 (71.6%)
**Cold ischemic time (hours)**	
Median [IQR]	6.00 [4.70,9.40]
Missing	63 (0.7%)
**Donor peak AST**	
Median [IQR]	114 [59.0,248]
Missing	18 (0.2%)
**Donor peak ALT**	
Median [IQR]	82.0 [39.0,189]
Missing	11 (0.1%)
**Donor peak creatinine (mg/dL)**	
Median [IQR]	1.30 [1.00,1.78]
Missing	20 (0.2%)
**Donor peak total bilirubin**	
Median [IQR]	0.800 [0.500,1.20]
Missing	10 (0.1%)

*Table S3: Multivariable cox model for early graft loss in the main cohort, pooled from 20 imputed datasets. Right-skewed variables not modelled with splines were log2-transformed, so the results relate to change every time the variable doubles. * for restricted cubic splines see Figure 3(C-D) and Figure S2. TTD = time to death; BMI = Body mass index; PDCA = pre-donation cardiac arrest; UW = University of Wisconsin; HTK = histidine-tryptophan-ketoglutarate; TX = Transplantation; MELD = Model for end-stage liver disease; HCC: Hepatocellular carcinoma; NASH = Nonalcoholic steatohepatitis; HCV = Hepatitis C virus; ICU = Intensive care unit; ALT = Alanine aminotransferase*

Variable	OR 95% CI	P value
*RCS Term: Time to Death	Wald test	0.149
NRP status		
SRR	Ref	-
NRP	0.503 (0.265 to 0.953)	0.035
Donor BMI (per 5 units)	1.098 (0.990 to 1.217)	0.077
Donor Sex		
Female	Ref	-
Male	1.036 (0.794 to 1.352)	0.795
Donor Hypertension History		
No	Ref	-
Yes	1.239 (0.915 to 1.677)	0.166
Donor Cause of Death		
Cerebrovascular/stroke	Ref	-
Anoxia with PDCA	0.709 (0.483 to 1.039)	0.078
Drug overdose	0.613 (0.382 to 0.984)	0.043
Head Trauma	0.725 (0.489 to 1.073)	0.108
Other	0.564 (0.333 to 0.955)	0.033
Static cold storage solution		
UW	Ref	-
HTK	1.316 (0.985 to 1.760)	0.063
Other	0.811 (0.568 to 1.158)	0.250
Machine perfusion		
None	Ref	-
Normothermic	0.574 (0.338 to 0.974)	0.040
Hypothermic	0.256 (0.032 to 2.031)	0.197
Unspecified	0.000 (0.000 to Inf)	0.992
Recipient Age (per 10 years)	1.117 (0.978 to 1.276)	0.101
Recipient BMI (per 5 units)	1.193 (1.073 to 1.326)	0.001
Recipient Sex		
Female	Ref	-
Male	1.014 (0.773 to 1.330)	0.919
MELD score (per 10 units)	1.110 (0.917 to 1.343)	0.285
Recipient Dialysis		
No	Ref	-
Yes	1.753 (1.005 to 3.060)	0.048
Recipient Diabetes		
No	Ref	-
Yes	1.160 (0.885 to 1.521)	0.282
Status1A		
No	Ref	-

Yes	5.916 (2.533 to 13.819)	<0.001
Recipient Primary Diagnosis		
Alcoholic Liver Disease	Ref	-
HCC	1.321 (0.887 to 1.967)	0.170
NASH	1.082 (0.705 to 1.661)	0.719
Cholestatic Disease	1.073 (0.567 to 2.028)	0.829
Acute Liver Failure	1.269 (0.495 to 3.251)	0.620
HCV	1.533 (0.939 to 2.500)	0.087
Other/Unknown	1.758 (1.179 to 2.621)	0.006
Recipient Medical Condition at TX		
Not Hospitalised	Ref	-
Hospitalised, but not in ICU	1.511 (1.037 to 2.201)	0.031
In ICU	3.232 (1.883 to 5.547)	<0.001
Recipient Functional Status (higher = better status)		
≤60%	Ref	-
70%	1.191 (0.874 to 1.623)	0.269
80%	0.845 (0.565 to 1.265)	0.414
90%	0.550 (0.238 to 1.271)	0.162
100%	0.264 (0.036 to 1.936)	0.190
Recipient Ethnicity		
White	Ref	-
Asian	0.828 (0.374 to 1.833)	0.642
Black	1.772 (1.152 to 2.727)	0.009
Hispanic	1.045 (0.739 to 1.480)	0.802
Other	1.605 (0.745 to 3.456)	0.227
Log2-Days on Liver Waitlist	1.079 (1.016 to 1.146)	0.014
*RCS Term: Donor Age	Wald test	0.028
*RCS Term: Donor Peak ALT	Wald test	0.218
*RCS Term: Transplant Year	Wald test	0.192
*RCS Term: Cold Ischemic Time	Wald test	0.170
*RCS Term: Admission to Donation Time	Wald test	0.005

*Table S4: Multivariable cox model for 1-year patient survival in the main cohort, pooled from 20 imputed datasets. Right-skewed variables not modelled with splines were log2-transformed, so the results relate to change every time the variable doubles. * for restricted cubic splines see Figure S3. Abbreviations: BMI, body mass index; PDCA, pre-donation cardiac arrest; TTD, time to death; HCC, hepatocellular carcinoma; NASH, nonalcoholic steatohepatitis; HCV, hepatitis C virus; AST, aspartate aminotransferase; ALT, alanine aminotransferase.*

Variable	HR 95%CI	P value
*RCS Term: Time to Death	Wald test	0.125
NRP status		
SRR	Ref	-
NRP	0.687 (0.443 to 1.066)	0.094
Donor BMI (per 5 units)	0.985 (0.913 to 1.064)	0.704
Donor Sex		
Female	Ref	-
Male	1.063 (0.881 to 1.283)	0.521
Donor Hypertension History		
No	Ref	-
Yes	1.210 (0.975 to 1.500)	0.083
Donor Cause of Death		
Cerebrovascular/stroke	Ref	-
Anoxia with PDCA	0.717 (0.549 to 0.936)	0.014
Drug overdose	0.712 (0.510 to 0.994)	0.046
Head Trauma	0.709 (0.542 to 0.929)	0.012
Other	0.648 (0.452 to 0.930)	0.019
Static cold storage solution		
UW	Ref	-
HTK	1.096 (0.885 to 1.356)	0.401
Other	0.852 (0.667 to 1.090)	0.202
Machine perfusion		
None	Ref	-
Normothermic	0.773 (0.539 to 1.108)	0.161
Hypothermic	0.033 (0.000 to 3.041)	0.139
Unspecified	0.549 (0.134 to 2.253)	0.405
Recipient Age (per 10 years)	1.316 (1.188 to 1.457)	<0.001
Recipient BMI (per 5 units)	0.947 (0.873 to 1.026)	0.183
Recipient Sex		
Female	Ref	-
Male	1.154 (0.949 to 1.402)	0.150
MELD score (per 10 units)	1.259 (1.098 to 1.444)	<0.001
Recipient Dialysis		
No	Ref	-
Yes	1.304 (0.852 to 1.997)	0.221
Recipient Diabetes		
No	Ref	-
Yes	1.333 (1.106 to 1.605)	0.002
Status1A		
No	Ref	-
Yes	3.144 (1.534 to 6.444)	0.002
Recipient Primary Diagnosis		
Alcoholic Liver Disease	Ref	-

HCC	1.451 (1.094 to 1.925)	0.010
NASH	1.512 (1.125 to 2.032)	0.006
Cholestatic Disease	0.864 (0.523 to 1.428)	0.568
Acute Liver Failure	1.218 (0.553 to 2.683)	0.624
HCV	1.356 (0.948 to 1.940)	0.095
Other/Unknown	1.715 (1.279 to 2.298)	<0.001
Recipient Medical Condition at TX		
Not Hospitalised	Ref	-
Hospitalised, but not in ICU	1.448 (1.114 to 1.883)	0.006
In ICU	2.152 (1.409 to 3.285)	<0.001
Recipient Functional Status (higher = better status)		
≤60%	Ref	-
70%	1.062 (0.850 to 1.327)	0.598
80%	0.845 (0.635 to 1.124)	0.248
90%	0.777 (0.466 to 1.296)	0.334
100%	0.931 (0.411 to 2.110)	0.864
Recipient Ethnicity		
White	Ref	-
Asian	0.604 (0.337 to 1.084)	0.091
Black	1.232 (0.872 to 1.740)	0.236
Hispanic	0.857 (0.663 to 1.108)	0.239
Other	1.845 (1.113 to 3.060)	0.018
Log2-Days on Liver Waitlist	1.064 (1.020 to 1.111)	0.004
*RCS Term: Donor Age	Wald test	0.834
*RCS Term: Donor Peak ALT	Wald test	0.645
*RCS Term: Transplant Year	Wald test	<0.001
*RCS Term: Cold Ischemic Time	Wald test	0.291
*RCS Term: Admission to Donation Time	Wald test	0.003

*Table S5: Multivariable cox model for recipient hospital length of stay in the main cohort, pooled from 20 imputed datasets. The HR is for discharge, so higher HR represents favorable outcome. Right-skewed variables not modelled with splines were log2-transformed, so the results relate to change every time the variable doubles. * for restricted cubic splines see Figure S4. Abbreviations: BMI, body mass index; PDCA, pre-donation cardiac arrest; TTD, time to death; HCC, hepatocellular carcinoma; NASH, nonalcoholic steatohepatitis; HCV, hepatitis C virus; AST, aspartate aminotransferase; ALT, alanine aminotransferase.*

Variable	HR 95%CI	P value
*RCS Term: Time to Death	Wald test	0.027
NRP status		
SRR	Ref	-
NRP	1.291 (1.181 to 1.411)	<0.001
Donor BMI (per 5 units)	0.994 (0.975 to 1.014)	0.557
Donor Sex		
Female	Ref	-
Male	0.984 (0.937 to 1.034)	0.520
Donor Hypertension History		
No	Ref	-
Yes	0.922 (0.871 to 0.976)	0.005
Donor Cause of Death		
Cerebrovascular/stroke	Ref	-
Anoxia with PDCA	1.068 (0.992 to 1.150)	0.081
Drug overdose	1.074 (0.984 to 1.173)	0.111
Head Trauma	1.072 (0.994 to 1.157)	0.072
Other	1.155 (1.051 to 1.269)	0.003
Static cold storage solution		
UW	Ref	-
HTK	0.932 (0.878 to 0.989)	0.020
Other	1.057 (0.996 to 1.123)	0.068
Machine perfusion		
None	Ref	-
Normothermic	1.179 (1.087 to 1.280)	<0.001
Hypothermic	1.192 (0.951 to 1.495)	0.127
Unspecified	1.644 (1.265 to 2.136)	<0.001
Recipient Age (per 10 years)	0.960 (0.937 to 0.983)	<0.001
Recipient BMI (per 5 units)	0.946 (0.927 to 0.966)	<0.001
Recipient Sex		
Female	Ref	-
Male	1.027 (0.977 to 1.080)	0.289
MELD score (per 10 units)	0.871 (0.839 to 0.904)	<0.001
Recipient Dialysis		
No	Ref	-
Yes	0.808 (0.684 to 0.954)	0.012
Recipient Diabetes		
No	Ref	-
Yes	0.935 (0.889 to 0.985)	0.011
Status1A		
No	Ref	-
Yes	0.532 (0.368 to 0.770)	<0.001
Recipient Primary Diagnosis		

Alcoholic Liver Disease	Ref	-
HCC	1.002 (0.933 to 1.076)	0.965
NASH	0.947 (0.880 to 1.020)	0.151
Cholestatic Disease	0.940 (0.849 to 1.040)	0.228
Acute Liver Failure	0.897 (0.702 to 1.145)	0.381
HCV	0.935 (0.850 to 1.028)	0.166
Other/Unknown	0.901 (0.835 to 0.972)	0.007
Recipient Medical Condition at TX		
Not Hospitalised	Ref	-
Hospitalised, but not in ICU	0.773 (0.716 to 0.834)	<0.001
In ICU	0.570 (0.491 to 0.662)	<0.001
Recipient Functional Status (higher = better status)		
≤60%	Ref	-
70%	1.142 (1.077 to 1.210)	<0.001
80%	1.209 (1.131 to 1.292)	<0.001
90%	1.193 (1.063 to 1.340)	0.003
100%	1.634 (1.318 to 2.025)	<0.001
Recipient Ethnicity		
White	Ref	-
Asian	0.848 (0.745 to 0.964)	0.012
Black	0.838 (0.756 to 0.929)	<0.001
Hispanic	1.024 (0.959 to 1.092)	0.481
Other	0.973 (0.834 to 1.135)	0.731
Log2-Days on Liver Waitlist	0.972 (0.961 to 0.983)	<0.001
*RCS Term: Donor Age	Wald test	0.023
*RCS Term: Donor Peak ALT	Wald test	0.891
*RCS Term: Transplant Year	Wald test	<0.001
*RCS Term: Cold Ischemic Time	Wald test	<0.001
*RCS Term: Admission to Donation Time	Wald test	<0.001

*Table S6: Multivariable cox model for 1-year graft survival in the main cohort with functional TTD (from 50mmHg), pooled from 20 imputed datasets. Right-skewed variables not modelled with splines were log2-transformed, so the results relate to change every time the variable doubles. * for restricted cubic splines see Figure S7. Abbreviations: BMI, body mass index; PDCA, pre-donation cardiac arrest; TTD, time to death; HCC, hepatocellular carcinoma; NASH, nonalcoholic steatohepatitis; HCV, hepatitis C virus; AST, aspartate aminotransferase; ALT, alanine aminotransferase*

Variable	HR 95%CI	P value
*RCS Term: fTTD 50mmHg (mins)	Wald test	0.581
NRP status		
SRR	Ref	-
NRP	0.520 (0.367 to 0.738)	<0.001
Donor BMI (per 5 units)	1.004 (0.946 to 1.065)	0.896
Donor Sex		
Female	Ref	-
Male	1.152 (0.992 to 1.339)	0.064
Donor Hypertension History		
No	Ref	-
Yes	1.311 (1.111 to 1.547)	0.001
Donor Cause of Death		
Cerebrovascular/stroke	Ref	-
Anoxia with PDCA	0.690 (0.561 to 0.850)	<0.001
Drug overdose	0.623 (0.477 to 0.814)	<0.001
Head Trauma	0.754 (0.611 to 0.930)	0.008
Other	0.599 (0.449 to 0.799)	<0.001
Static cold storage solution		
UW	Ref	-
HTK	1.050 (0.886 to 1.245)	0.572
Other	0.942 (0.782 to 1.136)	0.532
Machine perfusion		
None	Ref	-
Normothermic	0.546 (0.414 to 0.721)	<0.001
Hypothermic	0.167 (0.044 to 0.639)	0.009
Unspecified	0.510 (0.188 to 1.381)	0.185
Recipient Age (per 10 years)	1.067 (0.991 to 1.150)	0.086
Recipient BMI (per 5 units)	1.037 (0.975 to 1.102)	0.250
Recipient Sex		
Female	Ref	-
Male	1.197 (1.026 to 1.397)	0.022
MELD score (per 10 units)	1.128 (1.012 to 1.258)	0.030
Recipient Dialysis		
No	Ref	-
Yes	1.425 (1.001 to 2.028)	0.049
Recipient Diabetes		
No	Ref	-
Yes	1.266 (1.091 to 1.469)	0.002
Status1A		
No	Ref	-
Yes	3.039 (1.694 to 5.452)	<0.001
Recipient Primary Diagnosis		

Alcoholic Liver Disease	Ref	-
HCC	1.251 (1.007 to 1.555)	0.043
NASH	1.302 (1.038 to 1.633)	0.022
Cholestatic Disease	1.201 (0.860 to 1.676)	0.282
Acute Liver Failure	0.952 (0.502 to 1.804)	0.880
HCV	1.277 (0.964 to 1.692)	0.088
Other/Unknown	1.474 (1.177 to 1.847)	<0.001
Recipient Medical Condition at TX		
Not Hospitalised	Ref	-
Hospitalised, but not in ICU	1.254 (1.010 to 1.557)	0.040
In ICU	2.204 (1.568 to 3.098)	<0.001
Recipient Functional Status (higher = better status)		
≤60%	Ref	-
70%	1.195 (1.009 to 1.415)	0.039
80%	0.942 (0.761 to 1.165)	0.580
90%	0.759 (0.505 to 1.142)	0.186
100%	0.574 (0.255 to 1.292)	0.180
Recipient Ethnicity		
White	Ref	-
Asian	0.795 (0.520 to 1.215)	0.289
Black	1.250 (0.950 to 1.643)	0.111
Hispanic	0.900 (0.738 to 1.096)	0.295
Other	1.633 (1.090 to 2.446)	0.017
Log2-Days on Liver Waitlist	1.055 (1.020 to 1.091)	0.002
*RCS Term: Donor Age	Wald test	0.858
*RCS Term: Donor Peak ALT	Wald test	0.650
*RCS Term: Transplant Year	Wald test	0.026
*RCS Term: Cold Ischemic Time	Wald test	0.018
*RCS Term: Admission to Donation Time	Wald test	<0.001

Table S7: Exploratory analysis comparing donor instability between shorter TTD (<13 minutes) and longer TTD (≥13 minutes) donor cohorts. (A) Use of cardiovascular support (inotropes/vasopressors) at the time of donation. No significant differences in use between the cohorts. (B) Median values of respiratory support parameters in the last 24 hours before cross-clamp. No significant differences observed between cohorts. (C) Median values of pre-withdrawal observations in the 24 hours prior to cross-clamping. No meaningful differences were identified. pH = arterial blood pH; FiO2 = fraction of inspired oxygen; pAO2 = arterial partial pressure of oxygen; PEEP = positive end-expiratory pressure; RR = respiratory rate; SaO2 = arterial oxygen saturation; TV = tidal volume; BUN = blood urea nitrogen.

	TTD < 13mins (median, IQR)	TTD ≥ 13mins (median, IQR)
**A) Cardiovascular support**		
**Inotrope/Vasopressor use (% of cohort)**	3.49%	5.96%
**B) Respiratory support values**		
**pH**	7.415 (7.360-7.460)	7.425 (7.380-7.460)
**FiO2 (%)**	70.00 (40.00-100.00)	70.00 (40.00-100.00)
**pAO2 (mmHg)**	132.00 (92.0-241.50)	146.00 (99.00-283.50)
**PEEP (cmH2O)**	5.00 (5.00-8.00)	5.00 (5.00-8.00)
**RR (breaths/min)**	17.50 (14.00-20.00)	17.00 (14.00-20.00)
**SaO2 (%)**	98.40 (96.70-99.50)	98.70 (97.00-99.60)
**TV (ml)**	500.0 (433.0-540.0)	490.0 (430.0-520.0)
**C) Pre-withdrawal observations**		
**BUN (mg/dl)**	18 (12.5-28.0)	18 (12-26.5)
**Creatinine (mg/dl)**	0.85 (0.615-1.23)	0.80 (0.60-1.17)
**Sodium (mEq/l)**	146 (141-152)	146 (141-152)

*Table S8: Multivariable logistic model for utilization in the NRP cohort, pooled from 20 imputed datasets. Right-skewed variables not modelled with splines were log2-transformed, so the results relate to change every time the variable doubles. * for restricted cubic splines see Figure S11. TTD = time to death; BMI = Body mass index; PDCA = pre-donation cardiac arrest; ALT = Alanine aminotransferase.*

Variable	OR 95%CI	P value
*RCS Term: Time to Death	Wald test	<0.001
Donor Blood Group		
A	Ref	-
AB	0.385 (0.212 to 0.699)	0.002
B	0.773 (0.581 to 1.029)	0.077
O	0.980 (0.832 to 1.155)	0.812
Donor BMI (per 5 units)	0.849 (0.799 to 0.901)	<0.001
Donor Sex		
Female	Ref	-
Male	1.012 (0.855 to 1.196)	0.893
Donor Hypertension History		
No	Ref	-
Yes	0.942 (0.783 to 1.133)	0.524
Donor Diabetes History		
No	Ref	-
Yes	0.790 (0.647 to 0.965)	0.021
Donor Cause of Death		
Cerebrovascular/stroke	Ref	-
Anoxia with PDCA	1.321 (1.032 to 1.691)	0.027
Drug overdose	1.428 (1.018 to 2.002)	0.039
Head Trauma	1.270 (0.987 to 1.633)	0.063
Other	1.156 (0.876 to 1.526)	0.304
Machine perfusion		
No	Ref	-
Yes	7.434 (5.692 to 9.710)	<0.001
Donor Peak Serum Sodium	1.019 (1.009 to 1.028)	<0.001
*RCS Term: Donor Age	Wald test	<0.001
*RCS Term: Donor Peak ALT	Wald test	0.003
*RCS Term: Donor Peak Bilirubin	Wald test	<0.001
*RCS Term: Peak Albumin	Wald test	0.011
*RCS Term: Year of Donation	Wald test	<0.001

Supplementary figures

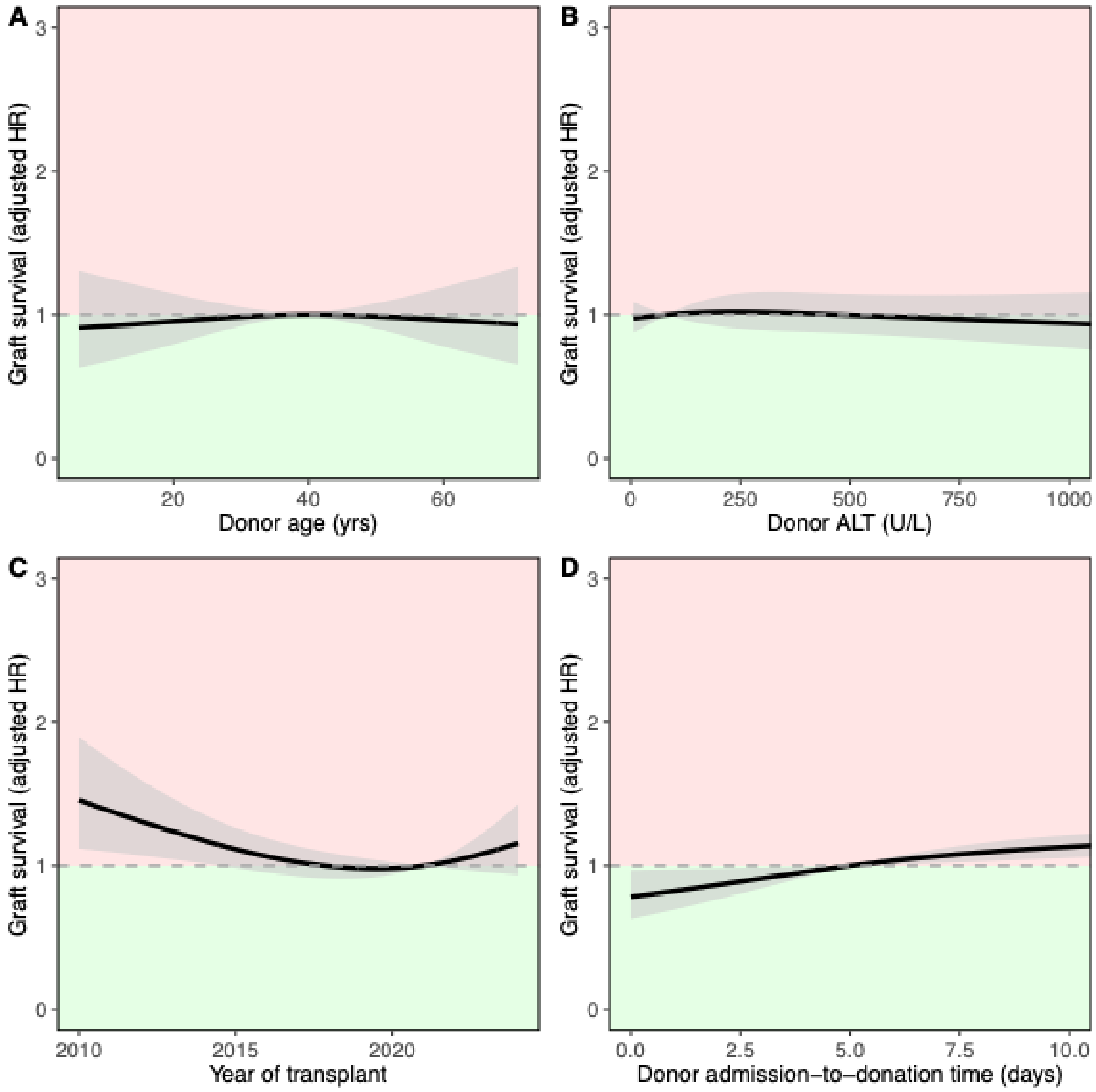

Figure S1: Impact on 1-year graft survival in the main cohort, adjusted for all of the factors shown in Table 2. Lines represent restricted cubic splines with grey shaded areas for 95% confidence intervals. Associations between (A) donor age, (B) donor ALT, (C) year of transplant, (D) donor admission-to-retrieval time against 1-year graft survival utilizing restricted cubic splines with 4 knots. These RCS models are derived from the model presented in Table 2. The green region represents superior outcome compared to the reference, while the red region signifies inferior outcome.

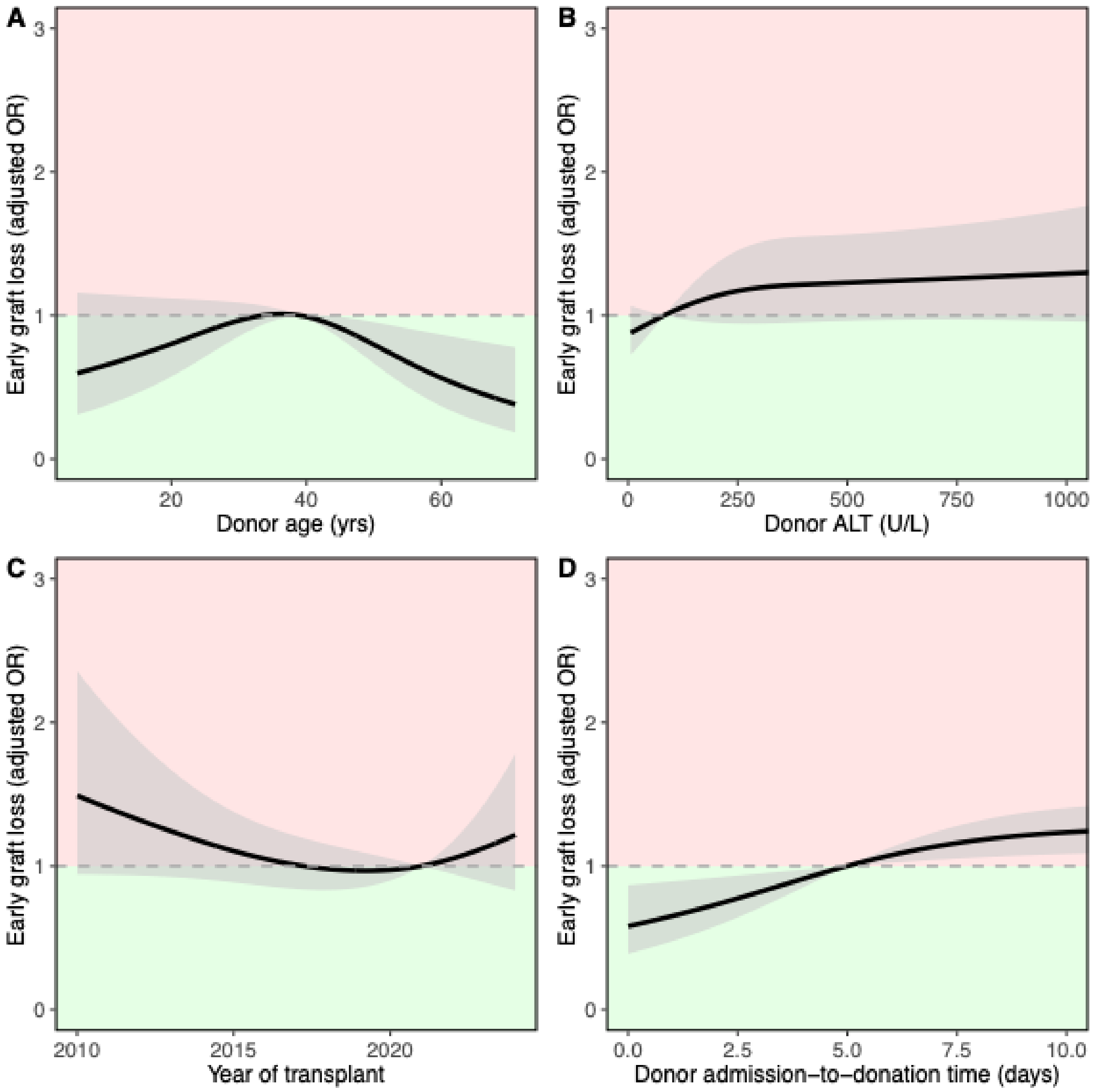

Figure S2: Impact on early graft loss in the main cohort, adjusted for all of the factors shown in Table S3. Lines represent restricted cubic splines with grey shaded areas for 95% confidence intervals. Associations between (A) Donor age, (B) donor ALT, (C) year of transplant, and (D) donor admission-to-retrieval time against early graft loss utilizing restricted cubic splines with 4 knots. These RCS models are derived from the model presented in Table S3. The green region represents superior outcome compared to the reference, while the red region signifies inferior outcome.

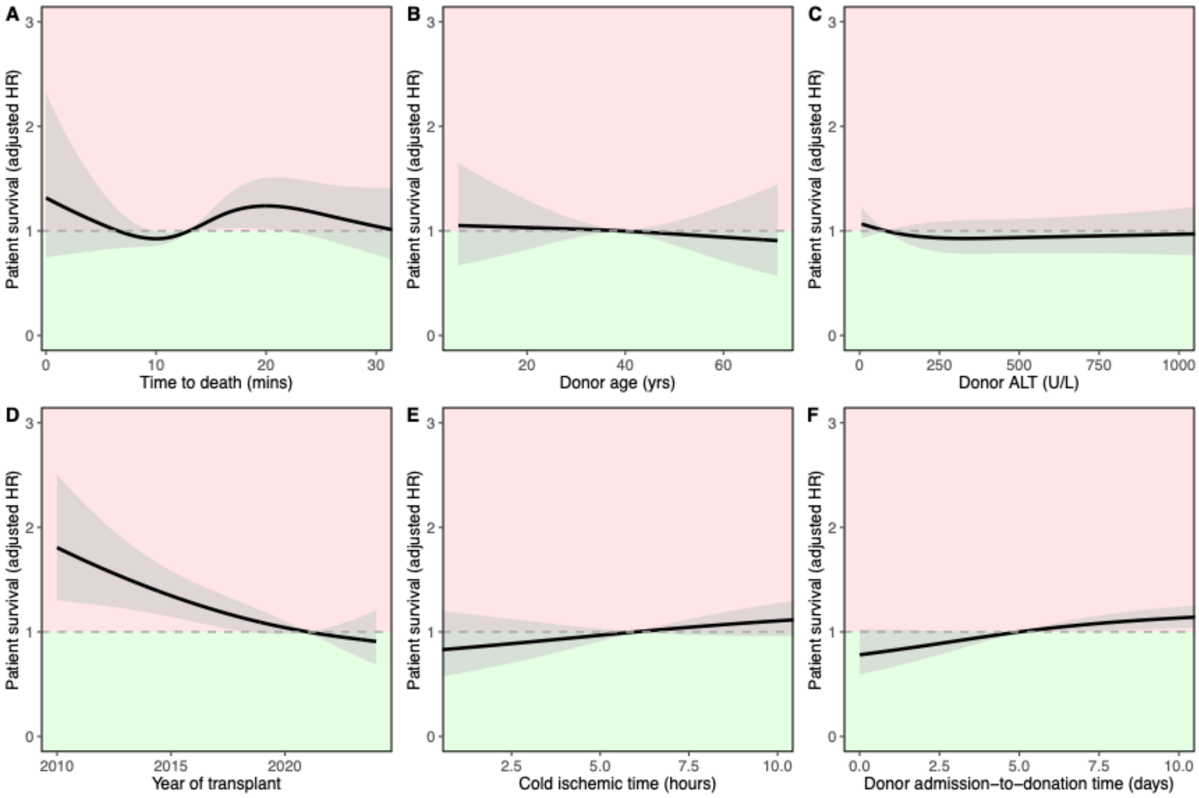

Figure S3: Impact on 1-year patient survival in the main cohort, adjusted for all of the factors shown in Table S4. Lines represent restricted cubic splines with grey shaded areas for 95% confidence intervals. Associations between (A) TTD, (B) donor age, (C) donor peak ALT, (D) year of transplant, (E) cold ischemic time, (F) donor admission-to-retrieval time against 1-year patient survival utilizing restricted cubic splines with 4 knots. These RCS models are derived from the model presented in Table S4. The green region represents superior outcome compared to the reference, while the red region signifies inferior outcome.

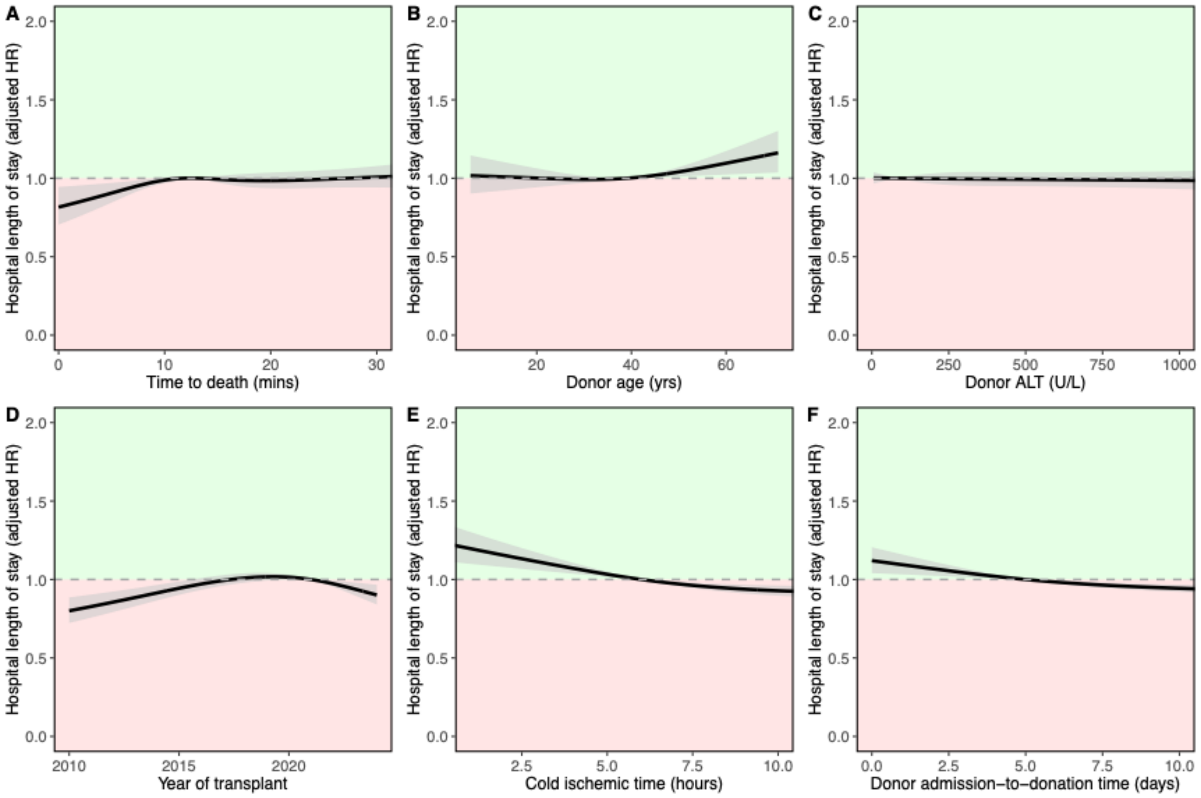

Figure S4: Impact on recipient hospital length of stay in the main cohort, adjusted for all of the factors shown in Table S5. Lines represent restricted cubic splines with grey shaded areas for 95% confidence intervals. Associations between (A) TTD, (B) donor age, (C) donor peak ALT, (D) year of transplant, (E) cold ischemic time, (F) donor admission-to-retrieval time against hospital length of stay utilizing restricted cubic splines with 4 knots. These RCS models are derived from the model presented in Table S5. The HR is for discharge, so higher HR represents favorable outcome. The green region represents superior outcome compared to the reference, while the red region signifies inferior outcome.

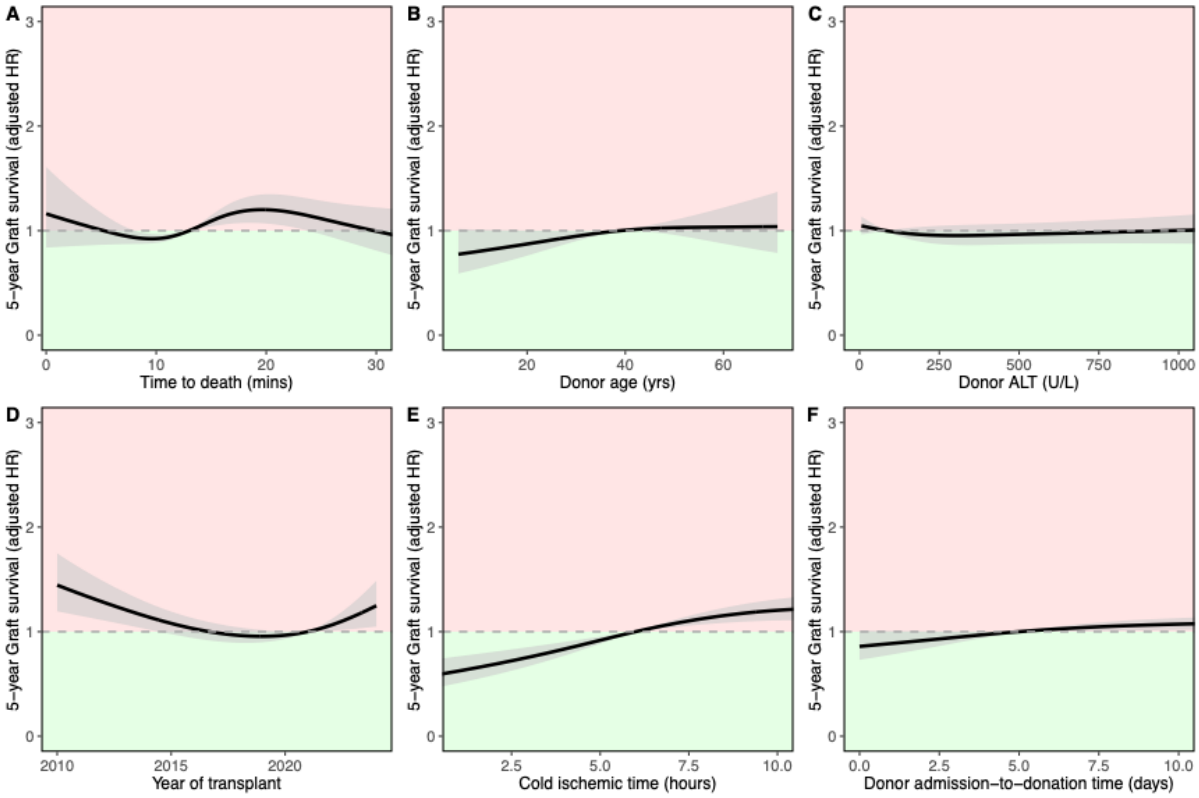

Figure S5: Impact on 5-year graft survival in the main cohort, adjusted for all of the factors shown in Table 2. Lines represent restricted cubic splines with grey shaded areas for 95% confidence intervals. Associations between (A) donor age, (B) donor age, (C) donor ALT, (D) year of transplant, (E) cold ischemic time, (F) donor admission-to-retrieval time against 5-year graft survival utilizing restricted cubic splines with 4 knots. The green region represents superior outcome compared to the reference, while the red region signifies inferior outcome.

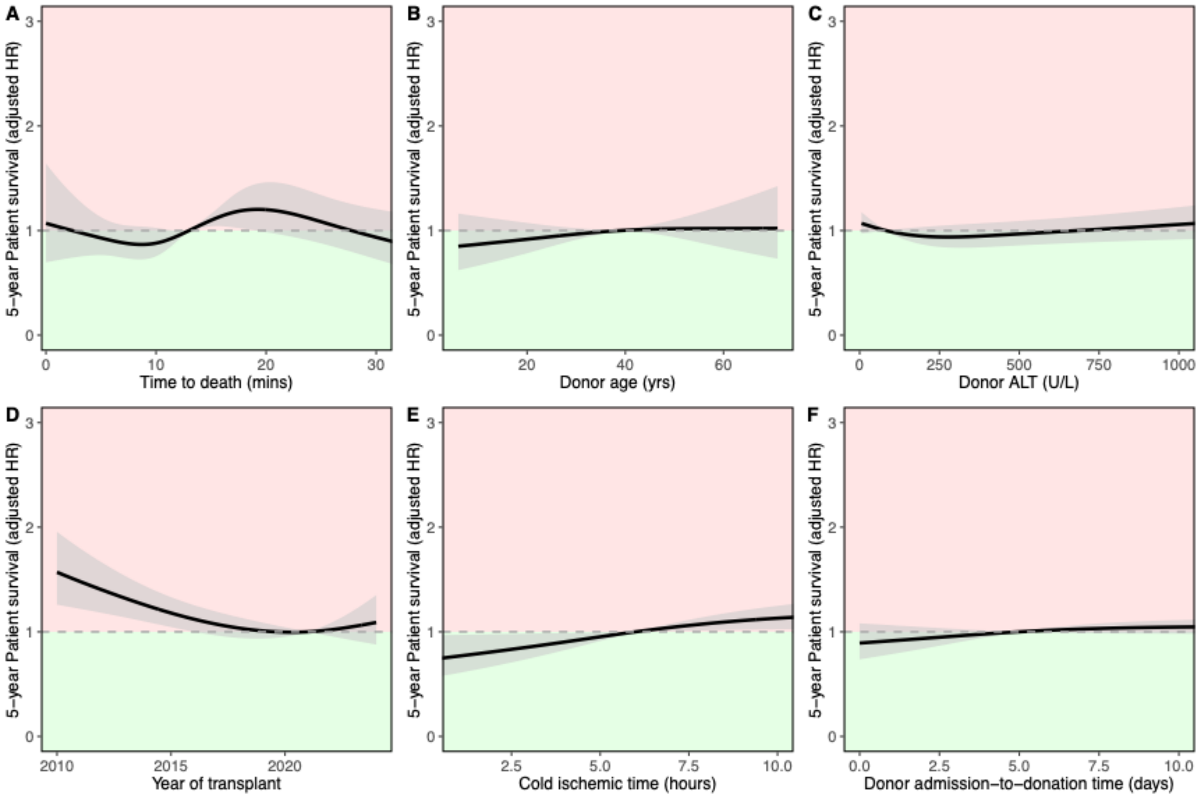

Figure S6: Impact on 5-year patient survival in the main cohort, adjusted for all of the factors shown in Table 2. Lines represent restricted cubic splines with grey shaded areas for 95% confidence intervals. Associations between (A) donor age, (B) donor age, (C) donor ALT, (D) year of transplant, (E) cold ischemic time, (F) donor admission-to-retrieval time against 5-year patient survival utilizing restricted cubic splines with 4 knots. The green region represents superior outcome compared to the reference, while the red region signifies inferior outcome.

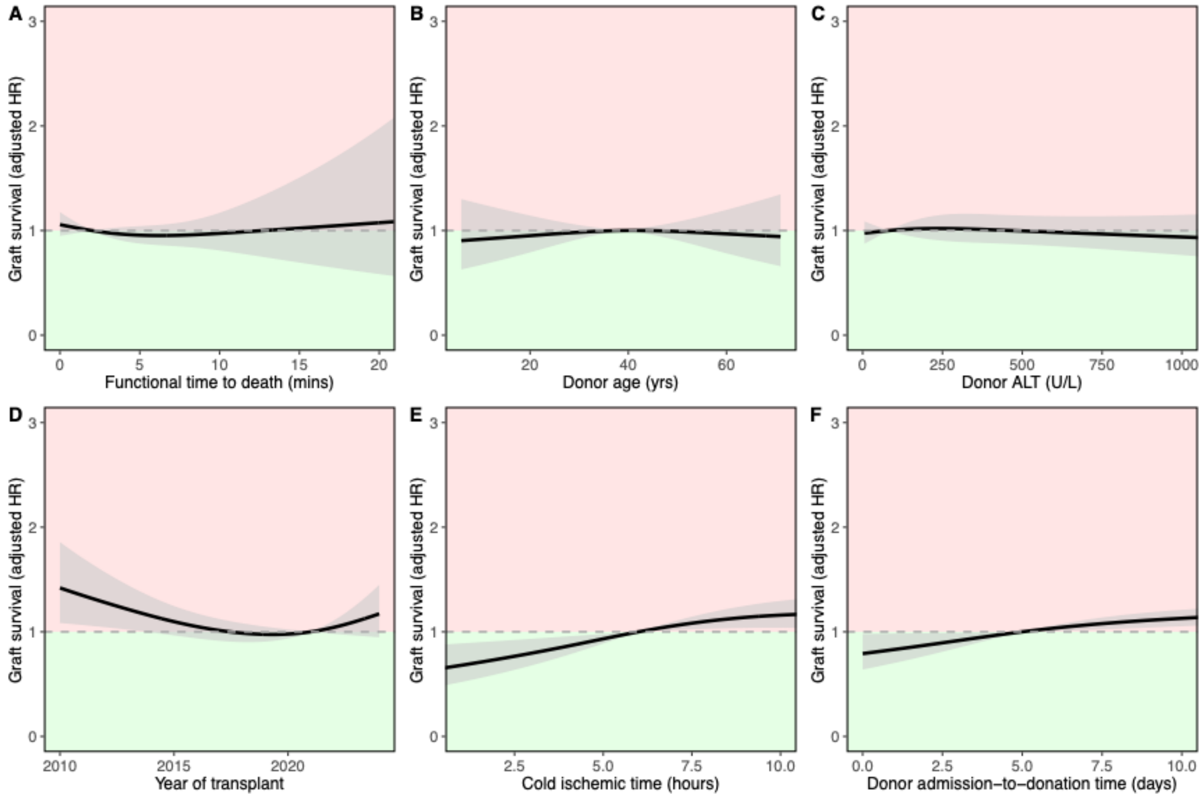

Figure S7: Associations between (A) FTTD (from 50mmHg), (B) donor age, (C) donor peak ALT, (D) year of transplant, (E) cold ischemic time, (F) donor admission-to-retrieval time against 1-year graft survival in the main cohort with fTTD from 50mmhg utilizing restricted cubic splines with 4 knots. These RCS models are derived from the model presented in Table S6. The green region represents superior outcome compared to the reference, while the red region signifies inferior outcome.

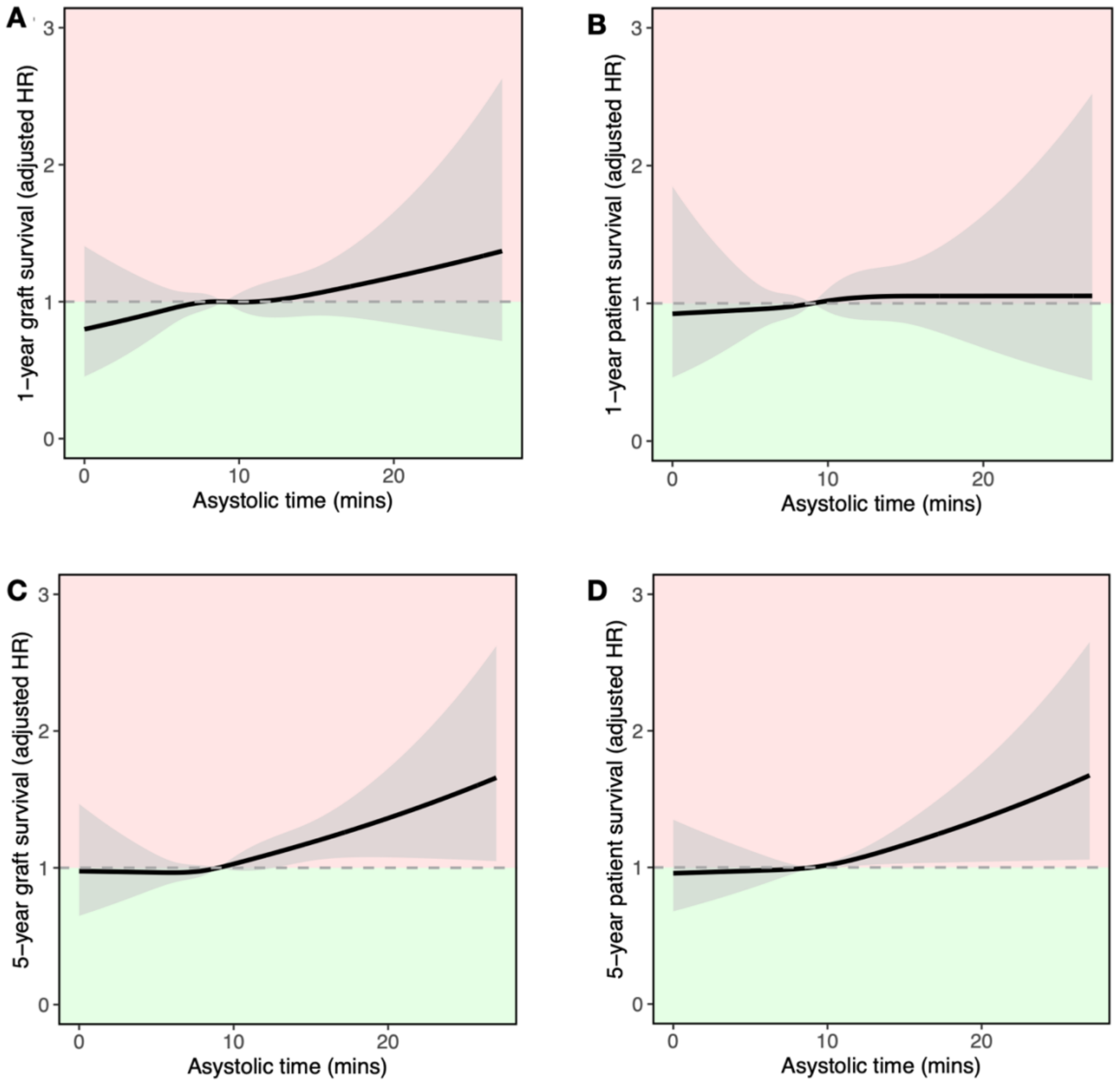

Figure S8: Impact of asystolic time on (A) 1-year graft survival, (B) 1-year patient survival, (C) 5-year graft survival, (D) 5-year patient survival, derived from models similar to TTD but also including asystolic time in the SRR cohort. Lines represent restricted cubic splines with grey shaded areas for 95% confidence intervals. The green region represents superior outcome compared to the reference, while the red region signifies inferior outcome.

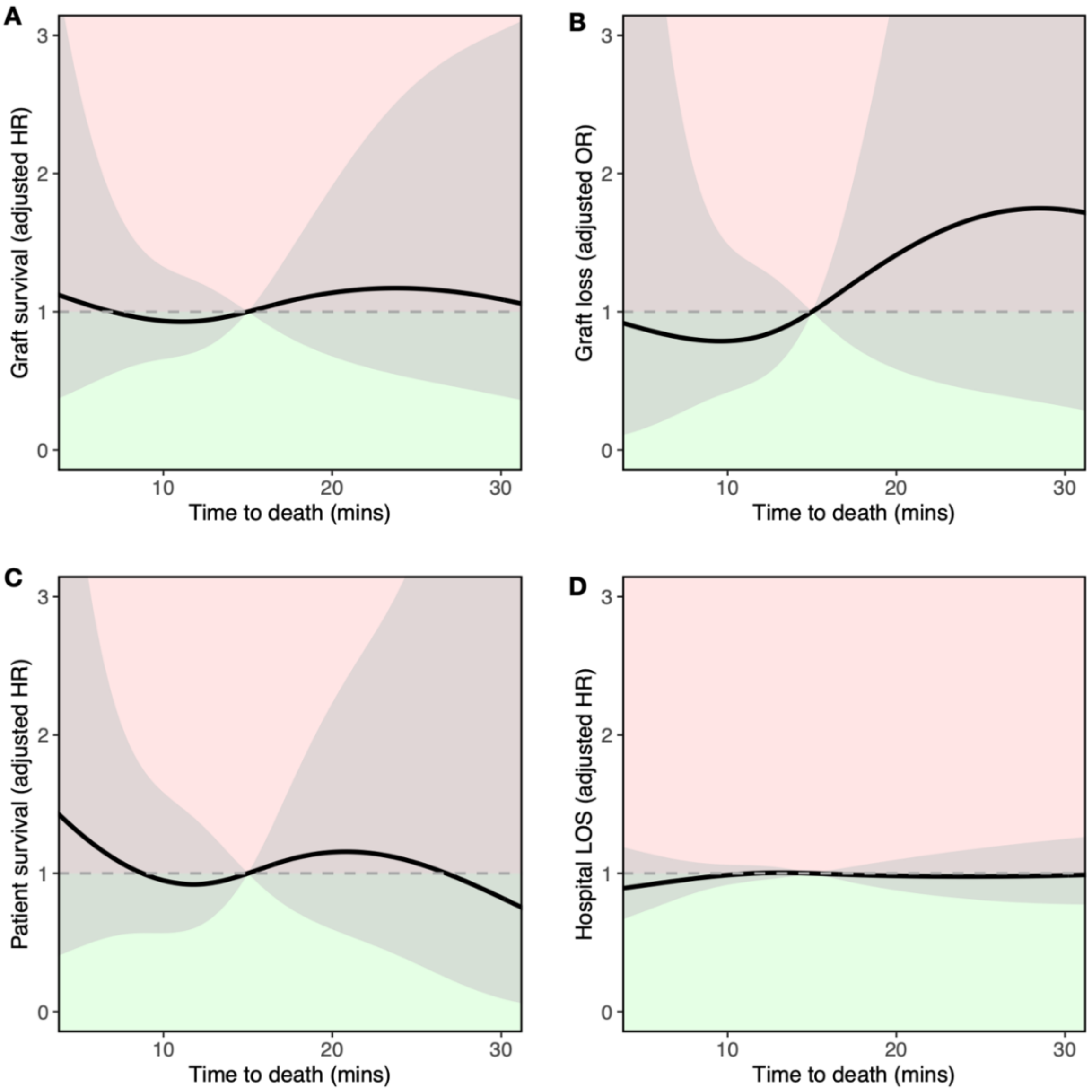

Figure S9: Association of TTD with (A) graft survival, (B) early graft loss, (C) patient survival, (D) hospital LOS, derived from models adjusting for machine perfusion, cold ischemic time and donor age in the NRP cohort. Lines represent restricted cubic splines with grey shaded areas for 95% confidence intervals. The green region represents superior utilization compared to the reference, while the red region signifies inferior utilization.

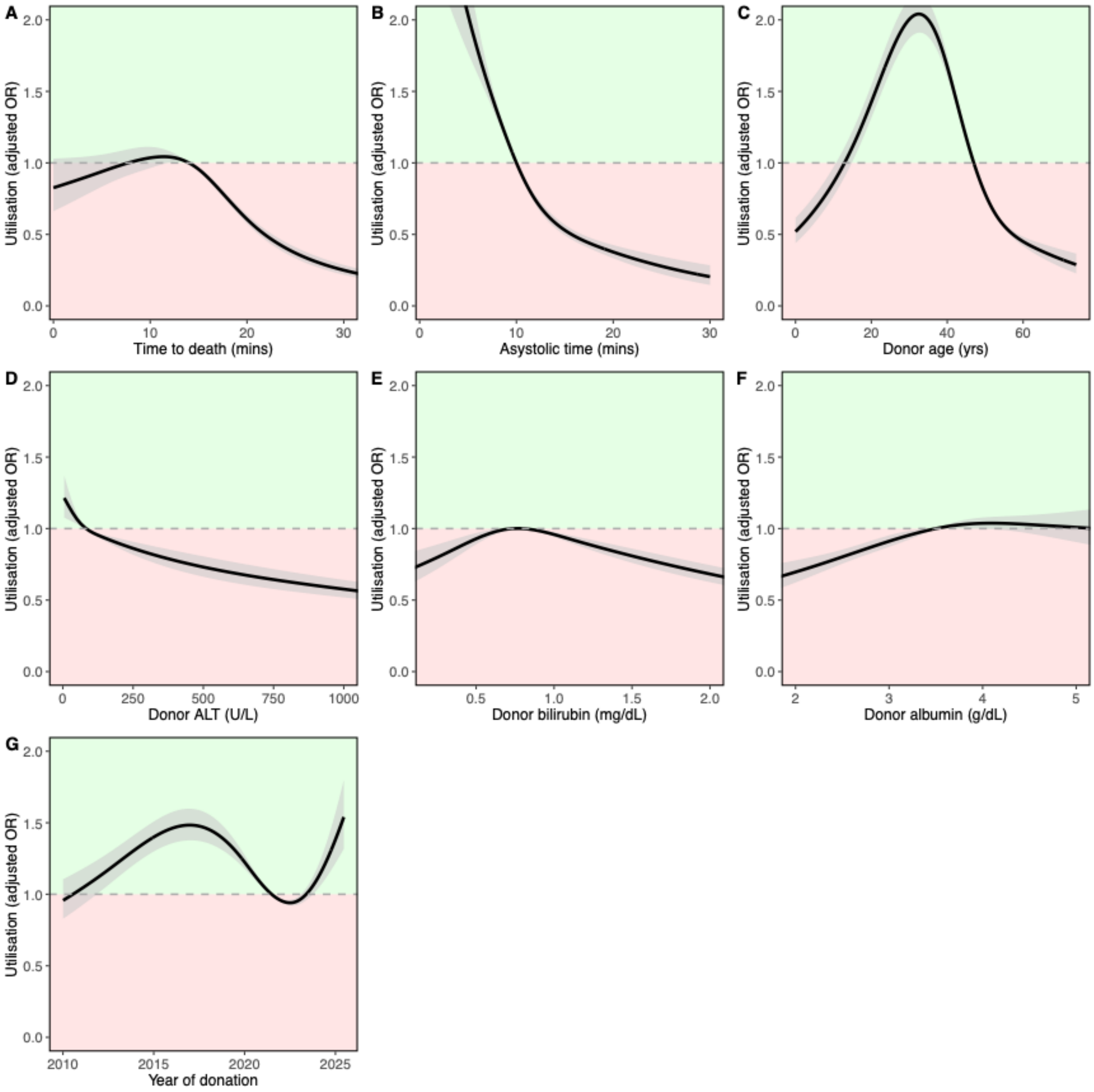

Figure S10: Impact of utilization using restricted cubic splines with 4 knots in the SRR cohort. Lines represent restricted cubic splines with grey shaded areas for 95% confidence intervals. Associations between (A) donor TTD, (B) asystolic time, (C) donor age, (D) donor ALT, (E) donor bilirubin, (F) donor albumin, (G) year of transplant against utilization in the SRR cohort. These RCS models are derived from the model presented in Table 3. The green region represents superior utilization compared to the reference, while the red region signifies inferior utilization.

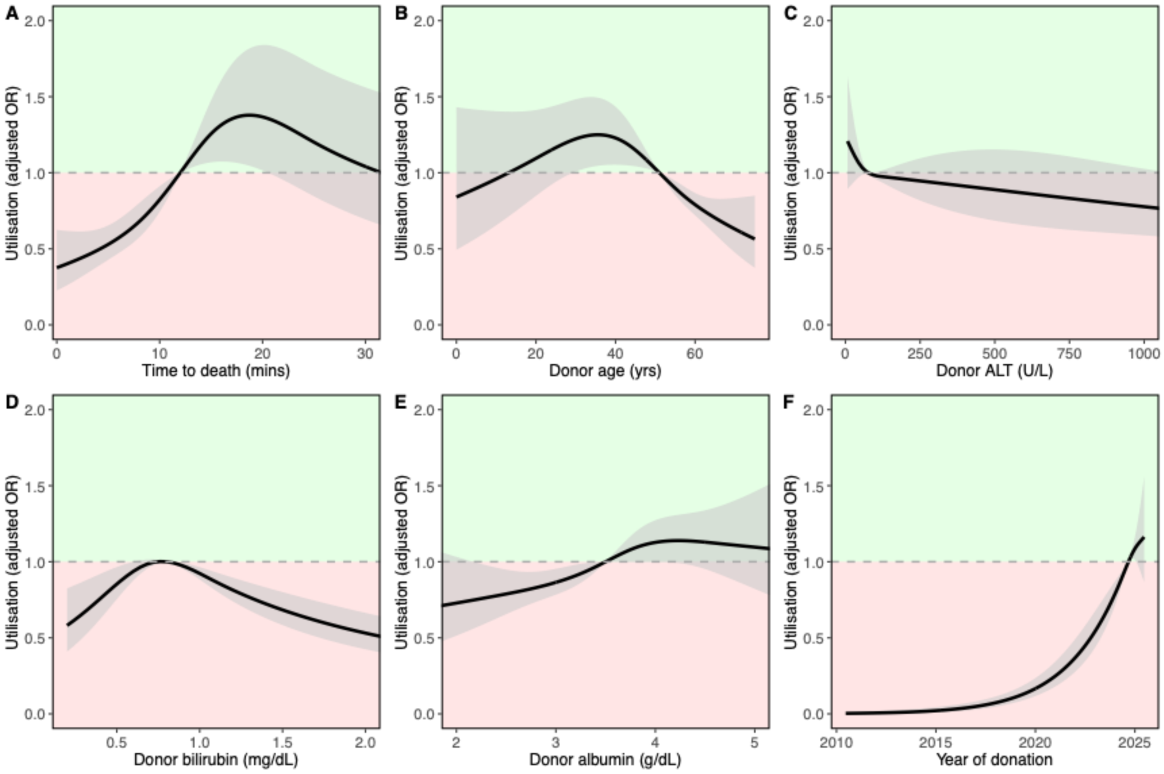

Figure S11: Impact of utilization using restricted cubic splines with 4 knots in the NRP cohort. Lines represent restricted cubic splines with grey shaded areas for 95% confidence intervals. Associations between (A) TTD, (B) donor age, (C) donor peak ALT, (D) donor bilirubin, (E) donor albumin, (F) year of transplant against utilization in the NRP cohort. These RCS models are derived from the model presented in Table S8. The green region represents superior utilization compared to the reference, while the red region signifies inferior utilization.
